# IoT-enabled multimodal large language model-based framework for compositional food recognition, nutrient profiling, NOVA classification, and cancer risk mitigation

**DOI:** 10.3389/fnut.2026.1970182

**Published:** 2026-10-26

**Authors:** Zebang He, Steve W. Y. Mung, Peggy Pui Lai Or, Chetwyn C. H. Chan

**Affiliations:** 1 Department of Science and Environmental Studies, The Education University of Hong Kong, Hong Kong, Hong Kong SAR, China; 2 Department of Health and Physical Education, The Education University of Hong Kong, Hong Kong, Hong Kong SAR, China; 3 Department of Psychology, The Education University of Hong Kong, Hong Kong, Hong Kong SAR, China

**Keywords:** digital healthcare, internet of things, large language model, NOVA classification, nutrition monitoring

## Abstract

**Background:**

Automated dietary assessment increasingly struggles to evaluate industrial food processing levels, revealing a systemic gap between the visual presentation of meals and their underlying ultra-processed food composition. Although existing dietary monitoring tools can effectively track caloric and macronutrient intake, they lack the qualitative processing assessments required to identify hidden additives and map dietary risk triage quadrants.

**Methods:**

This study introduces a modular, IoT-enabled multimodal dietary decision-support framework that decouples visual food recognition, standardized database retrieval, and qualitative language reasoning for automated NOVA processing classification and 2D decision matrix mapping. Operating on isolated single-dish meal instances, the system employs a four-phase cascaded pipeline that integrates vision-language models, database retrieval, and large language models (LLMs) with strict JSON schema enforcement. The platform was evaluated using the Nutrition-NOVA validation benchmark, a harmonized multimodal dataset constructed through dense vector-space semantic linkage between quantitative profiles from the Center for Food Safety database and qualitative NOVA tiers from Open Food Facts. This benchmark addresses a 75.0% metadata sparsity deficit identified in legacy repositories.

**Results:**

In experimental evaluations on the Nutrition-NOVA benchmark, the baseline Qwen-VL-Max architecture achieved an adjacent accuracy of 77.7%. Furthermore, enforcing schema modularity enabled complete plug-and-play backend replaceability between commercial cloud application programming interfaces (APIs) and private, on-premises open-weight models without requiring architectural retraining.

**Discussion:**

The proposed framework establishes an efficient, safety-first screening tool for digital healthcare decision support. By isolating qualitative LLM reasoning from visual perceptual noise and database metadata sparsity, the system successfully bridges the gap between intuitive meal perception and point-of-care dietary risk triage, offering a non-invasive and scalable solution for automated dietary auditing and longitudinal population health monitoring.

## 1 Introduction

Maintaining optimal dietary health has become the basis of modern preventative medicine and global public health initiatives (1). Although traditional nutritional science has primarily focused on controlling calorie intake and preventing basic micronutrient deficiencies, current digital health frameworks must address a remarkably complex metabolic landscape within personalized nutrition management (2). An individual's daily dietary intake directly influences cellular resilience, immune function, cardiovascular integrity, and long-term susceptibility to chronic diseases (3). Consequently, optimizing daily dietary patterns is no longer regarded merely as an individual lifestyle preference; rather, it represents an effective and non-invasive clinical intervention for reducing the increasing global burden of non-communicable diseases.

Despite the recognized importance of dietary quality and nutrition, modern nutritional landscapes are increasingly dominated by highly engineered industrial formulations that may pose severe physiological and pathological hazards due to their reliance on non-natural ingredients. Industrial food manufacturing regularly involves chemical extraction, aggressive thermal deconstruction, and the incorporation of complex chemical materials that can fundamentally alter the interaction between nutrients and the human gastrointestinal tract (4). Aiming to systemically measure and classify the level of food processing, public health researchers rely on the NOVA classification system, which categorizes all dietary entries into four distinct groups based on the extent and purpose of manufacturing (5). These categories range from Group 1 (unprocessed or minimally processed items), through Group 2 (culinary ingredients), Group 3 (standard processed foods), and Group 4 (ultra-processed foods, UPFs) (6). Isolating and classifying these processing levels is of critical clinical importance because prolonged exposure to Group 4 UPFs systematically breaks down natural food structures, alters metabolic responses, and exposes the body to neo-formed carcinogens produced during industrial food processing.

However, a systematic review of current digital health applications reveals a considerable gap in the state-of-the-art works (7). Although applications are available for calculating nutritional labels (8), and separate applications are designed to assess NOVA processing levels (9), no unified computational framework that combines both methodologies from everyday inputs is available for comprehensively evaluating personalized nutrition. Modern digital health applications are independent when evaluating the nutrition level of daily meals. Calorie-tracking tools mainly rely on a strictly quantitative paradigm to calculate calorific values, whereas NOVA-based applications focus exclusively on classifying food processing levels, completely failing to intersect the two. As illustrated by the traditional baseline paradigm in Figure 1, standard computer vision pipelines operate via a linear sequence encompassing food segmentation, ungrounded food volume estimation, and a final macronutrient calculation (10). However, once the food item is identified, traditional frameworks immediately diverge into two completely isolated computational streams:

- Isolated nutrition monitoring stream (left branch): This path attempts to follow a sequential pipeline that starts with food portion estimation and proceeds to the calculation of total nutritional values based on estimation portion (10). Forcing an AI to conduct 2D-to-3D geometric regression to estimate portion size from a single monocular image is highly unstable and can frequently lead to severe numerical hallucinations (11).
- Isolated food-processing-level stream (right branch): Similar to (9), this parallel path executes NOVA classification independently, operating completely detached from quantitative macronutrient profiling. Therefore, this path fails to integrate nutrient density into its risk assessment.

**Figure 1:**
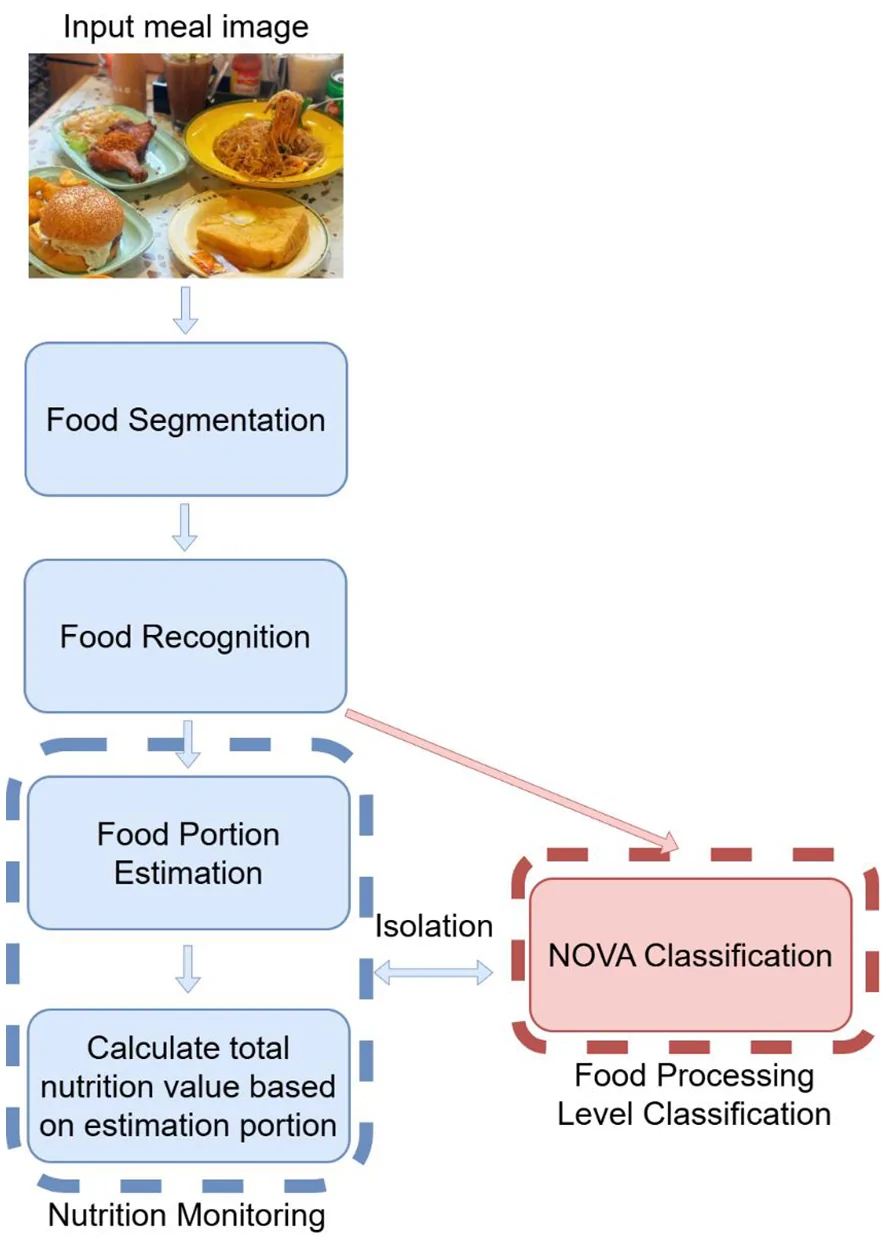
Block diagram of the traditional nutrition monitoring and food processing level classification application.

This structural separation introduces a critical double-blind spot. The nutrition stream emphasizes calorie estimation while remaining blind to hidden industrial additives, whereas the NOVA stream classifies processing tiers without integrating quantitative nutritional context. Consequently, a freshly prepared home-cooked dish and an engineered ultra-processed alternative may appear nutritionally equivalent within conventional calorie-tracking tools because chemical stabilizers, industrial emulsifiers, and hidden sugars are not visible under the vision-based pipeline (12).

The main motivation of this study stems from the recognition that an effective and reliable dietary diagnostic platform must bridge this gap by integrating open-world food item recognition with a dual-axis evaluation of baseline nutrition and food-processing quality. Independently evaluating either dimension creates massive health blind spots. For example, a commercial meal replacement or protein bar may appear clinically optimal on a standard nutrition label due to its high protein and low-fat content; however, a processing audit may expose the presence of industrial texturizers, cosmetic emulsifiers, and chemical flavor enhancers that contribute to its classification as a hazardous UPF. Conversely, foods naturally high in fat, such as salmon or raw nuts, might be penalized by a basic quantitative nutritional filter but validated as optimal by its non-UPF status. A multidimensional health profile can be constructed by intentionally integrating a food's nutritional profile with its processing level, thereby uncovering hidden biological risks without relying on flawed and unstable volumetric calculations.

Aiming to achieve this integration, a large language model (LLM)-based IoT-enabled framework is introduced. This framework shifts the paradigm of automated dietary tracking by employing multimodal LLMs as deep semantic reasoning engines. The proposed architecture eliminates unstable volume and portion-size estimation steps, routing the input meal image through a streamlined, four-phase retrieval–reasoning pipeline. In Phase 1 (multi-stage food recognition), the system performs food-region segmentation followed by region-based food classification, attempting to retrieve and match the identified food name directly against a verified regional repository, such as the Center for Food Safety (CFS) database. In the absence of exact database matches, an automated LLM recognition fallback loop is used. In Phase 2 (dual-path nutrition label profiling), the identified food items undergo dual-path verification. The system initially retrieves the nutrient label directly from the CFS repository, If the required information is unavailable, then it initiates retrieval-augmented generation (RAG) supported by external nutrition databases and LLM-based synthesis to establish a standardized baseline per 100 g. Concurrently, Phase 3 (automated NOVA processing classification) evaluates manufacturing quality through a parallel dual-path pipeline. The system queries the Open Food Facts (OFF) database (13) to retrieve explicit available processing tiers. In the absence of explicit processing information, an automated RAG and LLM-based text fallback layer is configured to parse ingredient structures and identify UPF signatures. Finally, Phase 4 (unified 2D decision matrix and risk quadrants) resolves the traditional separation in dietary information by routing the outputs into a 2D decision matrix that intersects nutrition information (high vs. low) against NOVA processing levels (UPF vs. non-UPF). This intersection maps the meal into an explicit quadrant verdict: a green light for healthy, nutrient-dense whole foods, a yellow light for transitional or disproportionate entries, and a definitive red light warning for energy-dense, nutrient-poor UPFs.

The contributions of this work are summarized as follows:

- Unified dual-axis evaluation framework: Introduces an end-to-end computational framework that combines quantitative chemical tracking (nutrition labels) and qualitative manufacturing taxonomy (NOVA level classification) from unconstrained meal imagery, resolving a historical separation in digital health informatics.
- Volumetric omission and hallucination mitigation: Through the systematic removal of error-prone 2D-to-3D portion and volume regression phases from the pipeline, the proposed framework replaces unstable geometric estimations with highly reproducible, standardized baseline profiling per 100 g, thereby eliminating numerical hallucinations.
- Hybrid dual-path knowledge retrieval: A robust retrieval–generation architecture that combines real-time visual grounding with verified localized public health databases and global nutrition databases is developed. This architecture is supported by an adaptive, zero-shot multimodal LLM fallback loop for complex and multi-cultural recipes.
- 2D matrix decision: A distinct four-quadrant decision matrix that intersects nutrient density against food processing tiers is formulated, thereby establishing a screening utility that maps food items into definitive green, yellow, and red diagnostic verdicts.

The remainder of this study is organized as follows. Section II presents an overview of the related literature, reviewing the historical development of computer vision-based dietary assessment and the emergence of food-processing classification frameworks. Section III details the comprehensive methodology of the proposed framework, focusing on the technical implementation of food recognition, hybrid RAG-based knowledge matching in nutrition label and NOVA classification level, and the construction of the 2D evaluation matrix. Section IV provides the experimental configurations, evaluation datasets, and benchmarking results compared against commercial LLMs. Finally, Section V concludes the paper and highlights future directions for automated digital preventative medicine.

## 2 Related work

### 2.1 Literature search strategy

A systematic literature search strategy was conducted across four primary digital repositories: PubMed/MEDLINE, IEEE Xplore, Google Scholar, and Scopus. The search architecture was systematically constructed by combining three distinct thematic query blocks using Boolean operators. The first block focused on advancements in artificial intelligence and computer vision, incorporating terms such as “Multimodal Large Language Models,” “Vision-Language Models,” “Deep Learning,” “Computer Vision,” and “Generative AI.” This block was combined with a second block isolating nutrition monitoring methodologies, which integrated terms including “Nutrition Monitoring,” “Dietary Assessment,” “Nutrient Profiling,” “Calorie Estimation,” “Volumetric Regression,” and “Nutrition Estimation.” The final block targeted food processing paradigms and incorporated keywords such as “NOVA Classification,” “Ultra-Processed Foods,” “UPFs,” “Food Processing Level,” and “Industrial Additives.”

The compiled literature was then screened using strict inclusion and exclusion criteria to ensure clinical and computational merit. Studies were explicitly included if they involve the following: peer-reviewed journal publications or landmark computer vision conference proceedings, including CVPR, ICCV, ECCV, and ACM Multimedia; and if they focused on open-world or multicultural food parsing or evaluated epidemiological links between dietary matrix degradation and oncological or metabolic clinical outcomes. Conversely, papers were systematically excluded if they documented proprietary commercial applications lacking architectural transparency, relied on restricted closed-world datasets featuring fewer than 20 distinct classes without real-world generalization, or omitted explicit statistical validation protocols against expert human benchmarks. This systematic approach ensured that the reviewed literature comprehensively captures the current intersection between artificial intelligence and nutritional science.

### 2.2 Traditional dietary and nutrition monitoring tool

Historically, dietary assessment in clinical environments and large-scale epidemiological cohorts has heavily relied on traditional self-reported instruments (14). These conventional tools primarily include 24-h dietary recalls (15), food frequency questionnaires (16, 17), and manual paper-based food diaries. Although these methods established the foundational data that supported the creation of early public health guidelines, they are still heavily limited by high patient cognitive load, low long-term compliance, and systematic recall bias, consistently leading to the underreporting of total energy intake (18, 19).

The transition to first-generation mobile health (mHealth) applications (20, 21) and digital dietary-logging tools streamlined this process through the incorporation of keyword-searchable national nutrient repositories, such as the USDA FoodData Central database (22). However, these digital platforms remain entirely passive and structurally dependent on manual data entry (23, 24). Users are required to accurately estimate portion sizes in grams or manually parse complex, multi-ingredient recipes into basic raw entries. Consequently, these legacy systems frequently suffer from high user abandonment rates and data fragmentation, failing to establish a scalable framework for continuous personalized clinical monitoring.

### 2.3 AI and computer vision in nutritional assessment

Aiming to eliminate the bottlenecks associated with manual logging, the intersection of computer vision and automated dietary assessment has become a crucial focus within biomedical informatics (25). Early deep learning approaches deployed deep convolutional neural networks (CNNs) (26) and vision transformers (27), primarily trained on closed-world classification datasets, to automate the identification of top-level food categories. Standard vision pipelines rapidly evolved into complex, multi-phase geometric estimation frameworks to extract explicit nutritional information. The standard architectures attempt to execute a rigid linear sequence involving the following: food pixel segmentation, spatial boundary region prediction, volumetric regression, and the application of estimated volume by a fixed density constant to output total macronutrient and calorie estimates (10).

Aiming to address the inherent scale ambiguity of basic 2D images, recent state-of-the-art improvements have introduced advanced depth map prediction methods (28, 29) and active sensory hardware (30, 31). Modern frameworks utilize specialized depth-prediction modules, frequently driven by vision transformers as encoders, to recover missing 3D spatial dimensions from a single monocular image by generating high-resolution depth maps and conducting cross-modal RGB-D feature fusion (32). Concurrently, hardware-driven approaches utilize smart mobile devices equipped with active time-of-flight LiDAR sensors, passive stereovision setups, or structured-light emitters to directly capture full-surface topologies (33). By eliminating the need for physical reference markers in the scene and drastically improving surface volume estimation accuracy, these methods convert visual food frames into high-fidelity 3D point clouds.

However, despite these technical advancements in geometric reconstruction, these systems still exhibit critical limitations in diagnostic accuracy when deployed in real-world environments. Monocular depth maps and hardware-based LiDAR point clouds are strictly surface-bound; thus, they suffer from severe occlusion challenges and fail to penetrate solid surfaces to detect multi-layered configurations. Therefore, hidden ingredients in common composite meals—such as burgers, lasagna sheets, deep bowls, or mixed stews—may remain undetected or inaccurately measured. More fundamentally, the continued reliance on depth and volume estimation reinforces a reductionist view of human nutrition that treats dietary quality purely as a function of physical volume and nutrition density. Even with highly accurate point-cloud reconstruction, these systems remain completely blind to qualitative food-processing transformations and hidden chemical modifications. Consequently, these methods may classify a fresh, home-cooked whole-food dish and an engineered, preservative-dense industrial alternative as structurally identical if they possess similar visual boundaries, highlighting the inability of current AI models to effectively track true metabolic and carcinogenic dietary risks.

#### 2.3.1 Emerging paradigms

From Geometric Estimation to System-Level Precision Nutrition. To overcome these reductionist visual boundaries, recent reviews articulate a paradigm shift toward data-driven, system-level AI integration in food science and clinical nutrition (34, 35). As highlighted by Leung et al. (34), contemporary food informatics reconceptualizes foods not as static macronutrient aggregates, but as dynamic biochemical matrices whose health impacts are governed by industrial manufacturing transformations. This computational paradigm necessitates moving beyond surface image classification toward structured knowledge representations—such as Knowledge Graphs and Semantic Ontologies—that map processing conditions and additive matrices into interpretable computational containers (34).

Concurrently, Agrawal et al. (35) emphasize that bridging personalized precision nutrition with smart food manufacturing requires adaptive AI frameworks capable of integrating multimodal computer vision, deep learning, and real-time food composition data to track nutrient preservation, additive exposure, and industrial processing severity. Extending this to clinical practice, recent reviews in precision dietetics highlight how AI algorithms eliminate the guesswork of self-reported logging by converting digital meal frames into dynamic, actionable dietary profiles (36). Furthermore, as synthesized in (37), advanced precision nutrition relies on multimodal data integration—combining computer vision, compositional database mapping, and digital feedback loops to handle episodic, error-prone dietary inputs and translate them into robust clinical decision support. Our proposed framework directly operationalizes these system-level paradigms: by decoupling monocular visual recognition from qualitative LLM reasoning and database harmonization, the platform bridges visual meal perception with industrial processing (NOVA) auditing to deliver a scalable, point-of-care 2D decision matrix.

### 2.4 Industrial food processing and the NOVA framework

Similar to advancements in quantitative nutrition tracking, nutritional epidemiology has undergone a major shift in perspective, recognizing that the long-term physiological impact of food is influenced not only by its chemical macronutrients but also by its level of industrial transformation. This qualitative dimension was standardized through the NOVA classification system (5, 6), which categorizes foods into four distinct groups based on the extent and purpose of manufacturing. Extensive longitudinal studies and comprehensive umbrella reviews have established a clear, dose-dependent relationship between high consumption of NOVA Group 4 UPFs and severe clinical outcomes, including metabolic syndrome, type 2 diabetes, systemic low-grade gut inflammation, and accelerated all-cause mortality (38, 39).

More recently, preventative oncology research has increasingly examined the structural transformation of natural food matrixes during UPF production, identifying notable associations between ultra-processed patterns and elevated multi-site cancer mortality risks (40). The aggressive thermal and chemical alterations utilized in manufacturing UPFs can generate neo-formed carcinogens, such as acrylamides, heterocyclic amines, and polycyclic aromatic hydrocarbons, which are further exacerbated by the introduction of synthetic chemical preservatives such as nitrites in commercial formulations (41). Moreover, the systematic inclusion of industrial cosmetic additives, particularly chemical stabilizers and synthetic emulsifiers, may actively disrupt the mucosal barrier of the human gastrointestinal tract, alter the gut microbiome, and lead to persistent low-grade chronic inflammation. Prolonged exposure to these chemical and structural alterations may directly drive DNA damage, promoting early-onset gastrointestinal malignancies and conventional colorectal adenomas (42).

### 2.5 Deep learning in food processing level prediction

Given the clinical importance of tracking processing tiers, computer scientists have recently attempted to automate the classification of dietary processing levels. Contemporary state-of-the-art works in this area almost exclusively rely on language models (43, 44) or rigid, rule-based keyword matchers (45) applied strictly to textual information extracted from packaged consumer goods. Large-scale open-source platforms, such as the OFF database, successfully leverage crowdsourced, text-based ingredient lists to train deep neural networks and gradient-boosted ensembles to identify explicit industrial markers. By identifying substances of rare culinary use or food additives (46), these text-driven systems can automatically flag the respective NOVA processing tier of a product with high precision or use machine learning classifiers, such as FoodProX (9), to mathematically map nutrient-density vectors directly into processing probability classes.

However, these text-bound applications introduce a critical technological limitation when deployed in real-world settings. They are entirely dependent on availability of physical barcodes or clean, pre-printed commercial ingredient labels on packaged items. When applied to unpackaged, freshly prepared meals—such as restaurant dishes, home-cooked multicultural recipes, or regional artisanal items—these text-bound models completely fail.

Concurrently, computer vision researchers have attempted to infer dietary metrics directly from imagery, but these efforts have predominantly focused on geometric portion-size estimation and caloric regression. Advanced visual frameworks deploy generative adversarial networks for single-view image-to-energy mapping (47) or 3D object scaling to enable the reconstruction of physical plate volumes (48). Although these geometric techniques advance portion estimation, they suffer from a fundamental diagnostic limitation: they treat dietary quality purely as a physical volume calculation, remaining blind to hidden industrial texturizers, emulsifiers, and chemical processing signatures that do not alter surface visual topology.

Aiming to address the need for direct visual processing assessment, pioneering end-to-end image classifiers such as DeepNOVA (49) have deployed deep CNNs to predict NOVA tiers directly from food photography. Despite supporting the feasibility of visual processing classification, end-to-end vision models present substantial clinical vulnerabilities. Operating as black-box architectures, these models lack interpretability, failing to explain the rationale behind assigned processing tiers and struggling with intra-class visual ambiguities, such as distinguishing visually identical raw, poached, or ultra-processed deep-fried variants. Furthermore, these vision models operate in isolation from structured nutritional databases; thus, they cannot perform multidimensional clinical triage. Therefore, a modular, knowledge-grounded framework that bridges visual perception, verified nutritional retrieval, and qualitative LLM reasoning is necessary.

### 2.6 Methodological comparison and research motivation

Aiming to contextualize the technical contributions, Table 1 systematically compares the proposed multi-modal framework against current state-of-the-art methodologies across primary application focus, technical strengths, and operational limitations.

**Table 1:** Comparison of the proposed platform and previous work.

References	Focus	Strengths	Limitations
Proposed platform	Food recognition, nutrition label profiling, NOVA classification, and 2D decision matrix	• Multi-modal fusion of visual cues and text-based database mapping. • Resolves database sparsity (the 75% missing NOVA gap in OFF) using an LLM-in-the-loop fallback • Clinical 2D decision matrix (Nutrition vs. NOVA processing) prevents flat dietary advice	• Higher inference latency compared to lightweight single-task networks (e.g., small CNNs) • Relies on the foundational visual/semantic reasoning limits of underlying VLMs
Fang et al. (47)	Food portion estimation	• Estimates complex food volume and energy distributions from a single 2D image • Bypasses manual geometric modeling using generative image-to-energy mappings	• Extremely sensitive to occlusion and overlapping food items • High computational overhead for GAN training • Fails on irregular/unstructured foods such as mixed salads
Vinod et al. (48)	Food portion estimation	• Reconstructs high-fidelity physical 3D representations of plates • Achieves sub-millimeter scaling accuracy using physical reference markers	• Highly impractical for real-world user workflows because it requires physical reference markers such as coins and cards • Fails on non-rigid, amorphous food items such as mashed potatoes and soup
Sempionatto et al. (30)	Nutrition estimation	• Captures real-time, passive, continuous intake behavior such as chewing and swallowing • High biometric correlation	• Extremely invasive hardware setups with poor long-term user adherence • Fails to determine the chemical, qualitative nutrient composition or NOVA processing tier of ingested meals
Han et al. (32)	Nutrition estimation	• Leverages depth maps to calculate true visual volume • Fuses physical depth with semantic segmentation masks for raw foods	• Highly sensitive to plate patterns, camera angles, and lighting glare • Completely unavailable to detect chemical processing levels, treating UPFs and whole foods with identical macronutrient profiles as equivalent
Menichetti et al. (9)	Nova classification	• Direct chemical-level classification based on structured ingredient text strings • High accuracy on pristine, standardized industrial databases	• Strictly text-based, unable to process real-world food images • Entirely breaks down when facing unbranded, localized meals, restaurant preparations, or raw whole foods lacking standardized ingredient lists
Hu et al. (43)	Nova classification	• Fine-tuned language models provide ultra-fast text-based database parsing • High accuracy across multi-lingual structures	• Completely constrained by database completeness; fails and drops records when encountering the systemic 75% missing NOVA data rows in global databases like OFF • Blind to visual cues or custom plate configurations
Elbassuoni et al. (49)	Nova Classification	• End-to-end direct visual classification of food processing levels using deep CNNs • High speed on simple, single-item images	• Lacks clinical interpretability, black-box model cannot explain why a specific NOVA level is chosen • Fails on intra-class visual variations, unable to differentiate visually identical items such as raw vs. steamed fish
Chun et al. (50)	Smart food area–weight sensing and portion estimation	• Combines deep learning image segmentation with automated area–weight calibration for accurate portion weights • Scalable deployment for institutional dining areas and canteens	• Constrained to structured dining environments with hardware sensor setups • Blind to qualitative food matrix degradation and ultra-processed food signatures
Lo et al. (51)	IoT multiscale weighing and direct intake monitoring	• Direct physical weight sensing with BLE-centralized data aggregation eliminates manual logging • High-precision real-time mass tracking for multi-item meal trays	• Requires dedicated physical scale hardware • Unable to audit qualitative food processing tiers (NOVA) or detect hidden industrial additives visually
He et al. (52)	Dysphagia safety monitoring and IDDSI texture auditing	• Operationalizes MLLM as an “intelligent soft sensor” for pre-consumption IDDSI texture auditing • Integrates chain-of-thought and bi-directional safety logic to minimize choking and aspiration risks	• Specifically tailored to mechanical texture/rheological safety for dysphagia care rather than general-population industrial processing (NOVA) or multidimensional 2D risk matrices

As detailed in Table 1, state-of-the-art dietary monitoring methodologies substantially vary in their main application focus, technical strengths, and operational limitations.

In Table 1, (47) and (48) focus on visual food portion estimation, while (30) addresses passive biometric sensing through wearable hardware. Comparing visual portion estimation techniques, depth-prediction fusion in (32) is superior to (47) because it leverages monocular depth maps to calculate true surface volumes, whereas the generative single-view image-to-energy mappings in (47) are highly sensitive to item occlusion and amorphous food shapes. Conversely, (48) achieves sub-millimeter physical scaling accuracy through 3D object scaling. However, this approach is less practical for everyday home use because it requires users to place physical reference markers (such as coins or cards) on the dining table. In smart dining environments, (50, 51) completely eliminate portion volume hallucinations by integrating physical sensing mechanisms. These systems combine automated image segmentation with area–weight calibration (20) or multiscale BLE weighing scales (21). However, both systems remain physically constrained to institutional canteens and structured dining trays.

For food processing level classification, text-driven models (9, 43) achieve high precision when parsing structured ingredient lists from commercial databases, whereas (49) performs direct visual NOVA classification from meal photographs using deep networks. However, (9, 43) fail on unpackaged, home-cooked, or restaurant meals that lack pre-printed ingredient labels, while (43) encounters severe bottlenecks in terms of the 75% missing NOVA data rows in global repositories such as the OFF database (49). Addresses text dependency through direct image classification but operates as an uninterpretable black-box model that struggles with intra-class visual variations (such as distinguishing raw fish from processed deep-fried fish). Furthermore, although specialized MLLM frameworks such as (52) successfully operationalize vision-language reasoning for IDDSI dysphagia texture safety, (52) is specifically designed for mechanical choking and aspiration risks rather than general-population industrial processing (NOVA) or multidimensional 2D clinical risk triage.

Compared to all prior methodologies summarized in Table 1, the proposed platform successfully combines visual plate recognition, knowledge graph database mapping, and qualitative LLM reasoning. By standardizing nutrition per 100 g mass, the proposed system bypasses the volumetric regression errors of (30, 32, 47, 48), the hardware friction of (30, 50, 51), the database sparsity limitations of (9) and (43), and the black-box opacity of (19), establishing an interpretable, multidimensional screening utility at the point of care.

## 3 Proposed framework

### 3.1 Framework overview

The core technical objective of the proposed framework is to decouple visual meal analysis from unstable and error-prone 3D geometric regression, replacing it with a highly reproducible dual-axis decision matrix.

As illustrated in the unified system topology presented in Figure 2, the proposed architecture eliminates portion-size estimation and instead processes an unconstrained input meal image through a modular, four-phase retrieval–reasoning pipeline. The framework processes raw image pixels into structured and standardized food item vectors, which are subsequently used to query regional and international food-science repositories. By operating on a standardized nutrition metric per 100 g unit, the system guarantees mathematical stability and cross-platform reproducibility across four sequential phases, as shown in Figure 2:

- Phase 1: Food recognition (blue line box): This phase involves food region segmentation and open-vocabulary VLM prediction, utilizing an LLM named entity recognition (NER) and a fuzzy-search fallback layer to align visual descriptors in the absence of exact CFS database matches.
- Phase 2: Nutrition label profiling (green line box): This phase facilitates the retrieval of quantitative macronutrient profiles directly from regional database records, utilizing RAG-backed LLM synthesis to profile unlisted items per 100 g.
- Phase 3: NOVA classification (red line box): This phase has a similar operation to phase 2, querying OFF database nodes or activating an LLM food-science auditor to detect industrial additives and assign an explicit NOVA tier (Groups 1–4).
- Phase 4: Overall evaluation (purple line box): This phase enables the intersection of quantitative nutrient density (High vs. Low) against qualitative manufacturing severity (non-UPF vs. UPF) within a 2D matrix, generating definitive green (healthy), yellow (transitional/high-nutrition UPF), or red (high-risk UPF) dietary risk triage classifications.

**Figure 2:**
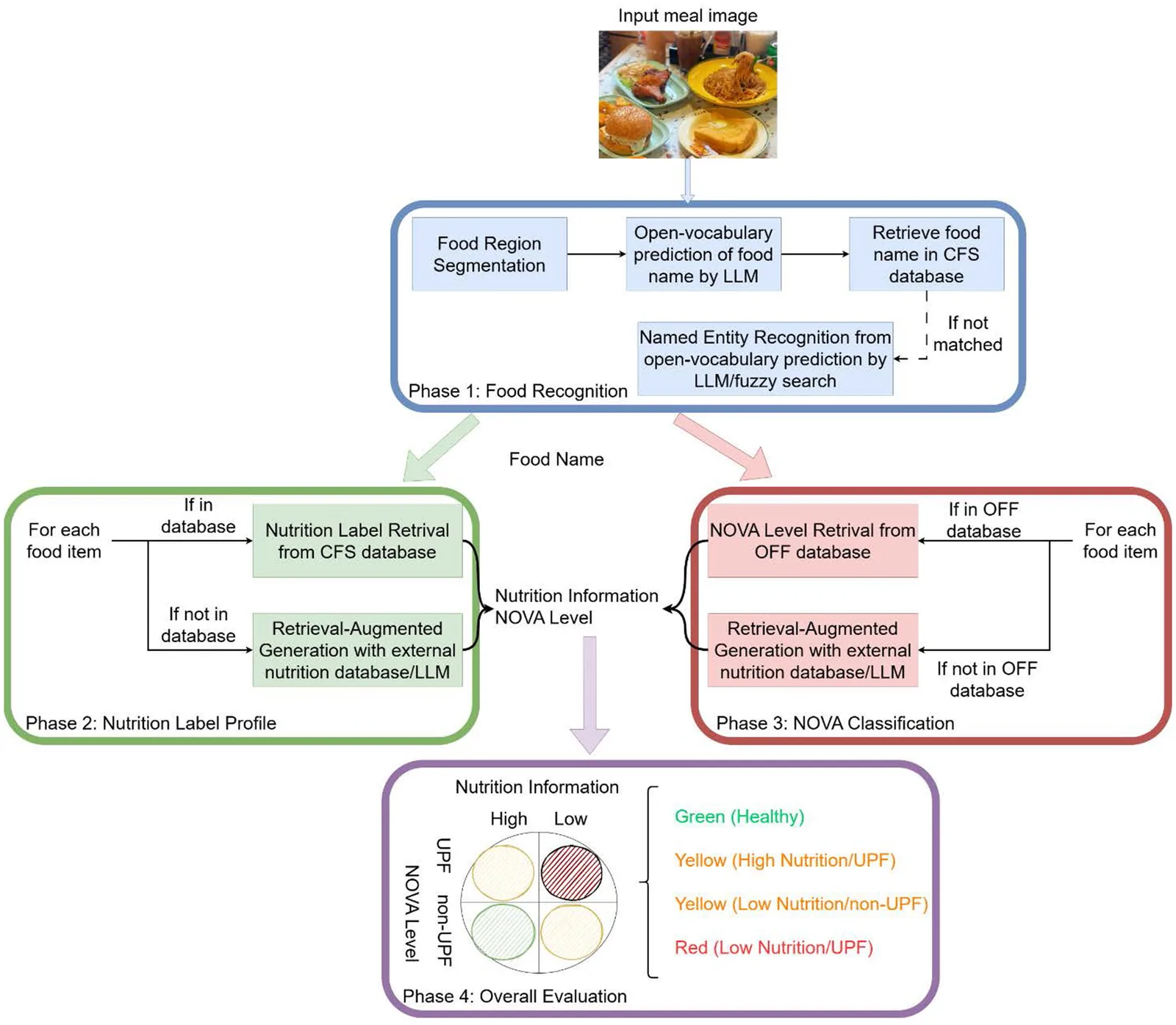
Workflow of the proposed framework.

### 3.2 Phase 1: multi-stage food recognition pipeline

As illustrated in the block diagram in Figure 3, Phase 1 transforms visual features extracted from a meal image into semantic text of food items, thereby bridging the gap between variable daily culinary presentations and rigid food database schemas. This phase utilizes a “predict–verify–align” execution loop to prevent pipeline failures caused by multi-cultural recipe naming variations.

**Figure 3:**
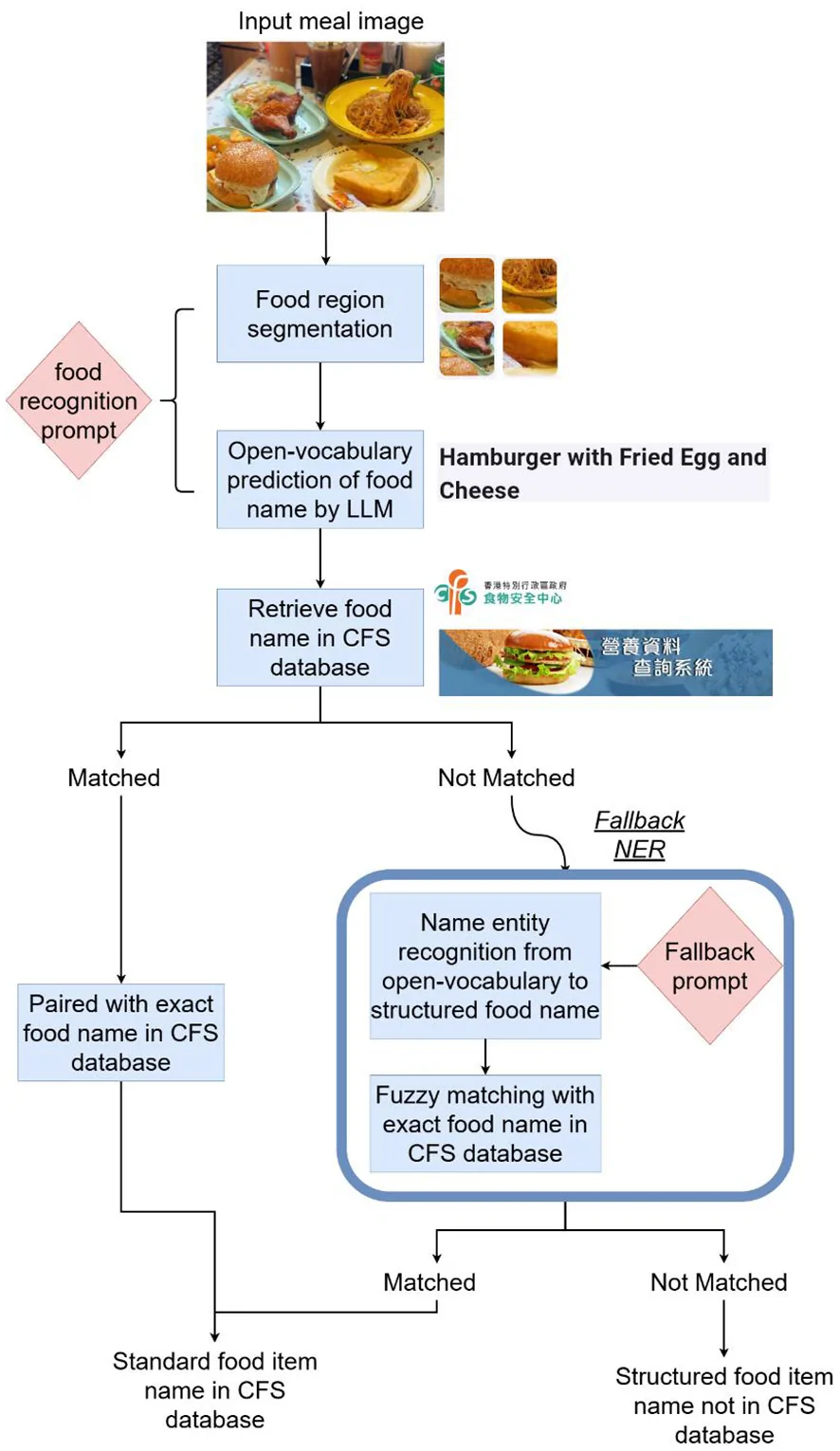
Block diagram of phase 1: multi-stage food recognition pipeline.

As depicted in Figure 3, the computational sequence begins when an unconstrained meal image is ingested by a multimodal LLM serving as the primary visual backbone. Guided by a specialized food-recognition prompt, the model first performs food region segmentation to identify distinct culinary elements on the plate and compute their spatial bounding boxes. These bounding boxes are then used to extract cropped image patches of individual food items (e.g., isolating individual portions, such as a burger, chicken leg, noodles, or toast).

Once these visual food items are isolated, the system triggers region-based classification through open-vocabulary food-name prediction by the LLM. In this sub-phase, the VLM performs zero-shot visual inference by evaluating localized textures, color spaces, and regional cooking configurations to generate an initial, highly descriptive open-vocabulary food-name string (e.g., “Hamburger with fried egg and cheese” or “Cantonese wok-fried soy sauce noodles with scallions”).

Once the raw visual description of each food item has been established, the framework initiates database verification through food name retrieval from the CFS database, querying the database using a strict string-matching filter. As shown in Figure 3, the workflow then evaluates two execution paths based on database matching results:

- Direct match path (matched): If an exact match occurs, then the item is paired with the corresponding food name in CFS database and immediately designated as a standard food item name. This verified food name is then passed directly to the downstream phases.
- Conditional fallback path (not matched): Owing to the highly descriptive nature of open-vocabulary MLLM outputs, exact string matching frequently fails against rigid database entries. Aiming to address these exceptions automatically, the framework routes unmatched text strings into a specialized fallback NER sub-module guided by a dedicated fallback prompt. During this step, the LLM functions strictly as a semantic compiler, executing NER to transform the open-vocabulary description into a structured food name, followed by fuzzy matching with the corresponding food names in the CFS database. This layer strips away non-essential culinary modifiers, isolates the core biological and structural ingredients, and aligns the description with its closest standardized equivalent.

As illustrated at the bottom of Figure 3, if this fuzzy alignment succeeds (matched), then the entity is output as a standard food item name in the CFS database; if unaligned (not matched), then the item is instead represented as a structured food item name not found in the CFS database. This process ensures a clean and standardized semantic payload for downstream nutrition label profiling and food processing level prediction.

### 3.3 Phase 2: dual-path nutrition label profiling

Upon receiving the validated food item names from Phase 1, the framework initiates Phase 2 to map the quantitative nutrient density of each identified food item. Figure 4 illustrates the complete computational workflow, from standardized food entity names to per-100 g nutrition labels. Aiming to maintain strict mathematical stability and eliminate errors associated with 2D-to-3D portion-size regression, all nutritional attributes—including total energy (kcal), proteins (g), carbohydrates (g), fats (g), calcium (mg), and iron (mg)—are normalized relative to a uniform reference mass of 100 g.

**Figure 4:**
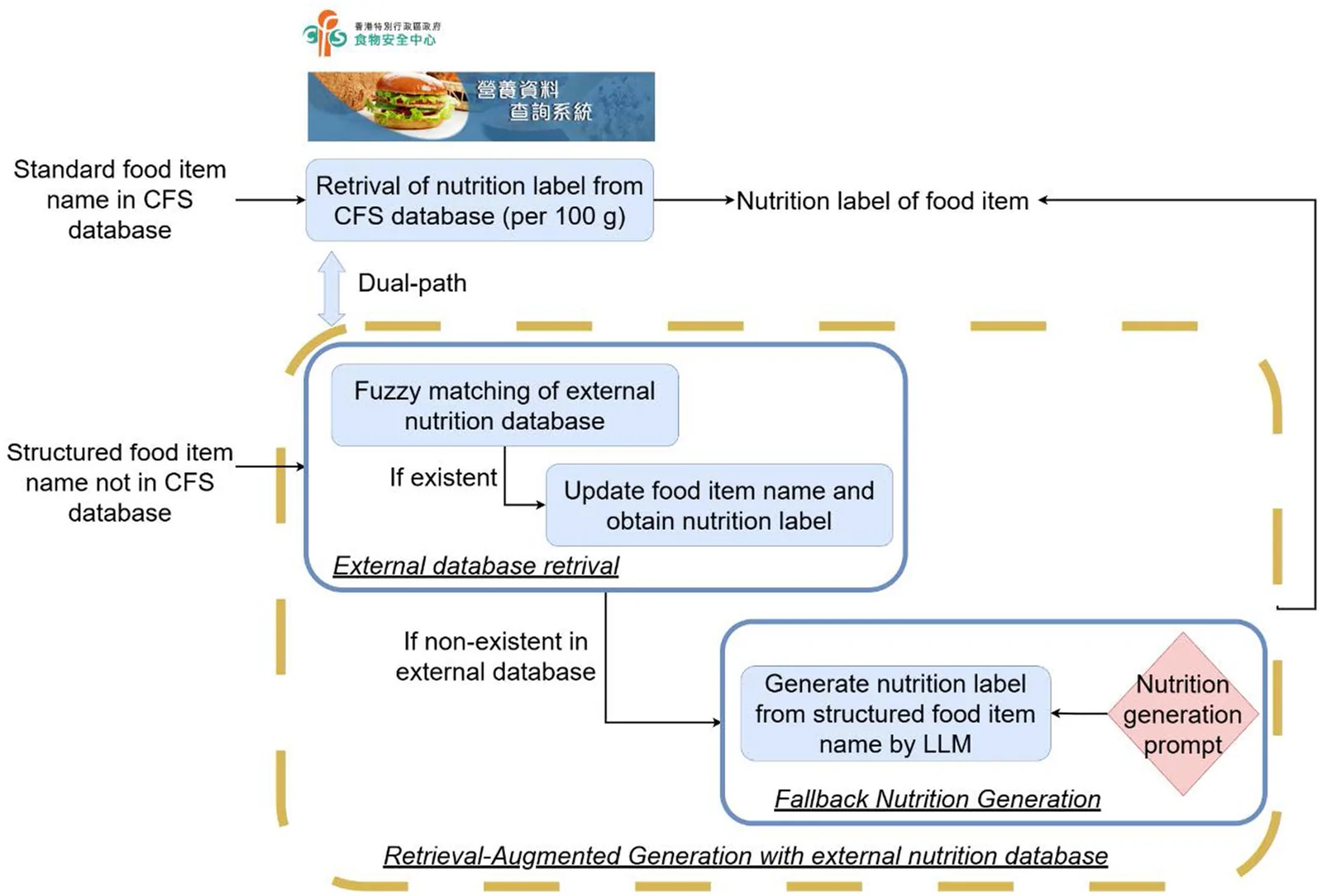
Block diagram of phase 2: dual-path nutrition label profiling.

As detailed in the block diagram in Figure 4, Phase 2 operates through a parallel, dual-path retrieval architecture to seamlessly handle missing database entries:

- Path 1: Direct CFS database retrieval (primary path): When the input token is a standard food item name in the CFS database, the system retrieves the corresponding nutrition label from the CFS database (per 100 g). This process directly returns verified ground-truth macronutrient and micronutrient values, which are used in the construction of the final nutrition label for the food item.
- Path 2: Retrieval-augmented generation with external nutrition database (secondary path): When the input token is a structured food item name not found in the CFS database, the system routes the entity to a RAG framework (dashed yellow box in Figure 4) comprising two sequential fallback mechanisms:

1. External database retrieval: The platform performs fuzzy matching against external nutrition databases and public health repositories [e.g., NHANES (53)]. If a match is found, then the system updates the food item name and obtains the corresponding nutrition label to populate the profile.
2. Fallback nutrition generation: If no match is found in the external databases, the unlisted item is passed to a text-based LLM guided by a specialized nutrition-generation prompt. The LLM generates a nutrition label from the structured food item by leveraging its parametric knowledge to synthesize a baseline per-100 g nutrient vector.

Aiming to illustrate the quantitative grounding mechanism of Phase 2, Table 2 presents representative nutritional profiles extracted from the CFS Knowledge Graph and harmonized with OFF entities, with all nutrient values normalized per 100 g mass across the four NOVA processing tiers (NOVA 1 to 4). As shown in Table 2, the database captures notable nutrient variations across processing levels—ranging from unprocessed whole foods (e.g., fresh raw whole egg and plum in Group 1) and processed culinary ingredients (e.g., sesame oil and sea salt in Group 2) to processed foods (e.g., shrimp paste and sweet vinegar in Group 3) and ultra-processed industrial formulations (e.g., fried shrimp and beer in Group 4).

**Table 2:** Selected samples of nutrition information from CFS.

Food name (CFS)	Food name (OFF)	NOVA level	Energy	Food name (CFS)	Food name (OFF)	NOVA level	Energy	Food name (CFS)
Beer	Beer	4	43	0.46	3.55	0	4	0.02
Fried shrimp (fast food style, breaded, fried)	Fried shrimps	4	277	11.51	24.39	15.18	51	1.8
Shrimp paste	Shrimp paste	3	100	15.2	0	4.3	667	8.7
Sweet vinegar	Sweet seasoned vinegar	3	37	2.8	6.5	0	42	9.7
Sea salt	Sea salt	2	0	0	0	0	49	2.6
Sesame oil (for salad or cooking)	Sesame oil	2	884	0	0	100	0	0
Fresh raw whole egg	Fresh eggs	1	143	12.57	0.78	9.94	53	1.83
Plum	Plum	1	46	0.7	11.42	0.28	6	0.17

Nutritional values are normalized per 100 g of food.

### 3.4 Phase 3: automated NOVA processing classification

Concurrently, the standardized food entity vectors generated in Phase 1 are passed to Phase 3 to evaluate the qualitative processing level of each food item. Figure 5 illustrates the complete computational workflow for NOVA classification based on standardized food item names. Operating similarly to the quantitative nutritional profiling phase, Phase 3 executes a dual-path logic within a RAG framework (dashed gold box in Figure 5) to resolve database sparsity and determine the processing level of each food item.

**Figure 5:**
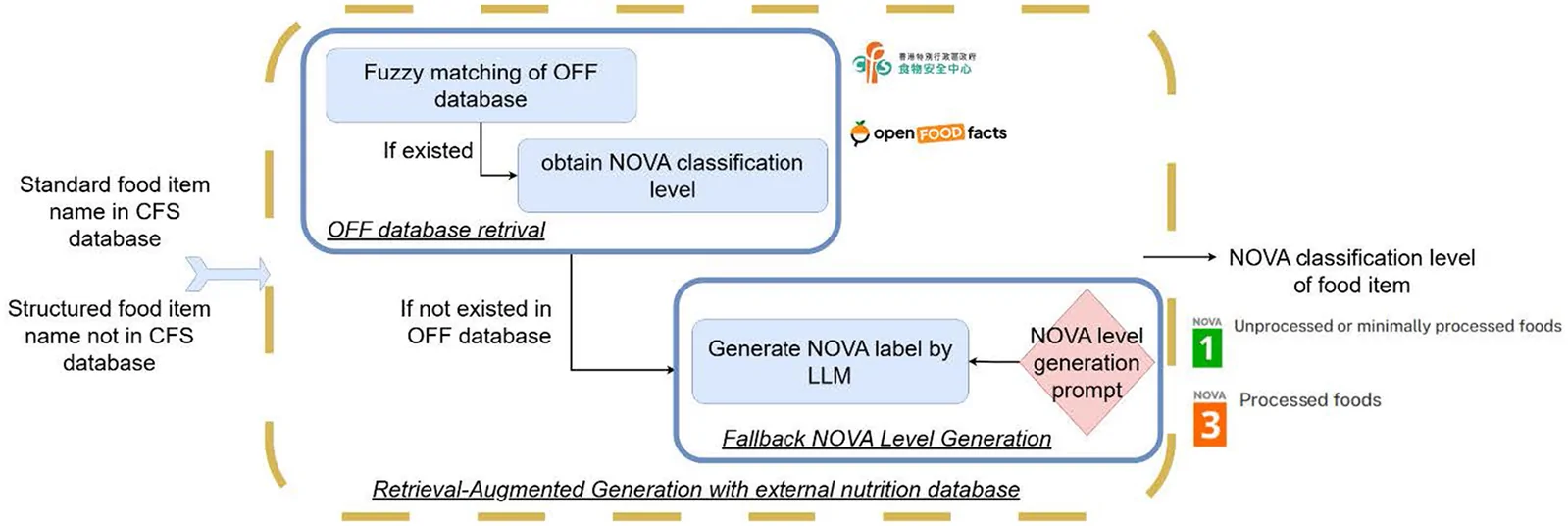
Block diagram of phase 3: automated NOVA processing classification.

As detailed in the block diagram in Figure 5, the execution flow processes standard food item name in the CFS database and structured food item name not found in the CFS database entries across two distinct sub-modules:

- Module 1: OFF database retrieval: The system first executes fuzzy matching against the OFF database to retrieve the historical processing tier of identical or semantically similar food items. If a match is found, the system obtain the NOVA classification level, directly mapping the entry to NOVA Groups 1, 2, 3, or 4.
- Module 2: Fallback NOVA level generation: If no match is found in the OFF database, the pipeline triggers a specialized LLM fallback. In this branch, the text-based LLM is guided by a NOVA level generation prompt to generate the corresponding NOVA label. The model functions as an expert food-science auditor, executing rule-based ingredient decomposition to identify markers of industrial processing. If the LLM detects substances that are rare in culinary preparations or characteristics of chemical additives—such as artificial texturizers, emulsifiers, high-fructose corn syrups, hydrogenated oils, or flavor enhancers—the model flags the ingredient matrix and assigns a qualitative tier of NOVA group 4 (UPFs).

The final output yields an explicit NOVA classification level for each food item, categorizing meals into four standardized tiers: NOVA Group 1 (unprocessed or minimally processed foods), NOVA Group 2 (processed culinary ingredients), NOVA Group 3 (processed foods), and NOVA Group 4 (ultra-processed food products).

### 3.5 Phase 4: unified 2D decision matrix and risk quadrants

In the final overall evaluation phase, the isolated streams of quantitative chemical data and qualitative processing metrics are integrated. As illustrated in the detailed mathematical and qualitative mapping schema in Figure 6, Phase 4 maps these dual-axis outputs onto a novel 2D decision matrix that intersects chemical nutrient density with the severity of industrial manufacturing.

**Figure 6:**
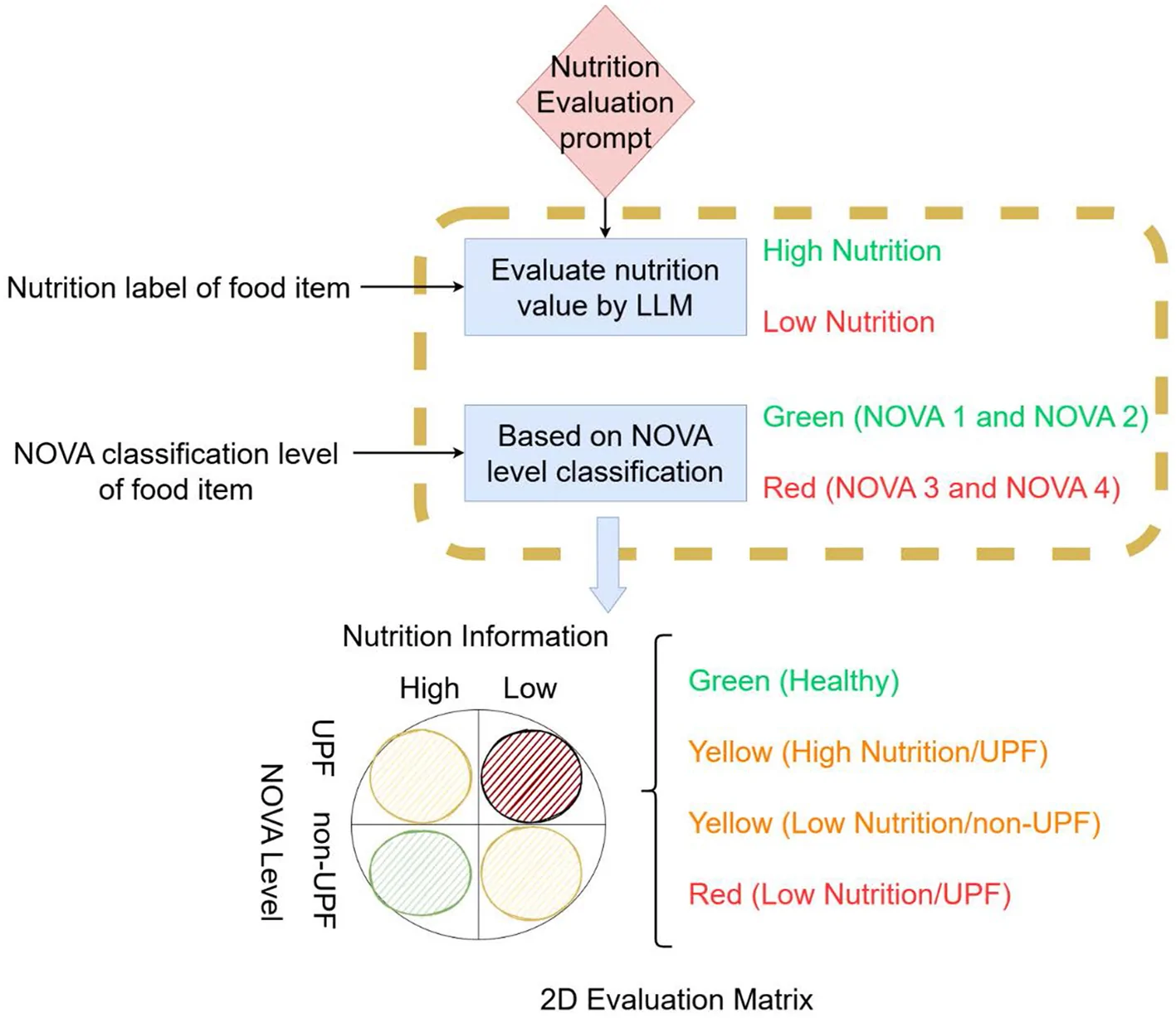
Block diagram of phase 4: unified 2D decision matrix and risk quadrants.

As shown in the block diagram (dashed gold box in Figure 6), the integration workflow receives the nutrition label for each food item from Phase 2 and the NOVA classification level for each food item from Phase 3:

•  Evaluation of nutritional value using LLM (*Nut)*: Guided by a dedicated nutrition evaluation prompt, the text-based LLM evaluates the macronutrient and micronutrient vectors to classify nutrient density. To eliminate subjective ambiguity, nutrients are not weighted equally. Instead, the prompt enforces a standardized Nutrient Profiling System schema (54) evaluating nutrients per 100g reference mass across two weighted categories:

° Positive / Qualifying Nutrient Vector: Protein, dietary fiber, calcium, and iron contribute positive points (+1 to +5 per threshold).
° Negative / Risk Nutrient Vector: Energy density (kcal/100g), saturated fat, free sugars, and sodium incur penalizing negative points (−1 to −5 per threshold).

LLM would imply the composite nutrient score from positive and negative nutrient vector to automatically get *Nut* = “0” or “1,” where “1” denotes high nutrient density and “0” denotes low nutrient density.

- Classification based on NOVA level: The pipeline maps the NOVA processing tier to a binarized manufacturing tier, categorizing NOVA Groups 1, 2 as Green and NOVA Groups 3, 4 as Red. Similarly, the qualitative processing tier is binarized as *Nova* = “0,” “1,” where “0” represents non-proceed food corresponding to NOVA Groups 1 and 2, while “1” represents processed foods or UPFs under NOVA Groups 3 and 4.

The framework then evaluates the overall health-risk profile using a 2D matrix, which assigns each meal into one of four distinct diagnostic quadrants, as defined below:


$$\begin{array}{l} Diagnosis\;Result=\\ \left\{ \begin{array}{l} \;Green,\;if\;Nut\text{​}=\text{​}1\;and\;Nova=0\\ \text{​}Yellow,\;if\;(Nut=1\;and\;Nova\text{​}=\text{​}1)\;or\;(Nut=\;0\;and\;Nova\;=0).\\ \;Red,\;if\;Nut=0\;and\;Nova=1 \end{array}\right.\text{​} \end{array}$$ (1)

As shown in the circular 2D Evaluation Matrix at the bottom of Figure 6, the intersection of these dual axes categorizes meal items into four distinct diagnostic quadrants. The green light quadrant (*Nut* = “1” and *Nova* = “0”) designates healthy, nutrient-dense whole foods that actively support metabolic resilience. The first yellow-light quadrant (*Nut* = “1” and *Nova* = “1”) isolates foods that appear nutritionally sound on paper, such as high-protein bars or processed shakes, but may pose potential concerns due to industrial additive risks. The second yellow-light quadrant (*Nut* = “0” and *Nova* = “0”) identifies minimally processed foods with low nutrient density but remain free of synthetic chemical adulteration, such as pure culinary sugars or fats. Finally, the red-light quadrant (*Nut* = “0” and *Nova* = “1”) flags highly engineered industrial formulations that exhibit low nutritional density alongside high ultra-processing levels, highlighting items strongly associated with long-term metabolic health risks in epidemiological literature. An easy-to-use nutrition screening utility is established by transforming food health classifications into a visible 2D decision matrix.

## 4 Experiment and result analysis

Aiming to rigorously validate the multimodal dietary analysis framework, a multi-tiered empirical evaluation was conducted. The framework decouples visual recognition from nutritional knowledge retrieval and qualitative processing reasoning through a four-phase architecture. This architecture utilizes vision-language models (VLMs) for visual food identification and spatial segmentation, nutrition database retrieval for standardized nutrient mapping, and LLMs for NOVA processing classification and the generation of a 2D dietary risk triage classification matrix. For experimental platform benchmarking, the framework was configured using the commercial Qwen API suite (Qwen-VL-Max and Qwen-Max, version 2026.07.30 snapshot) as the underlying computational backend.

### 4.1 Benchmark for dietary risk triage accuracy: the nutrition-NOVA validation benchmark

A novel, multimodal ground-truth dataset was used to rigorously assess the proposed 2D dietary decision framework. Existing public food databases typically exist in isolated siloes: some provide extensive quantitative macronutrient profiles [e.g., USDA (22), Food-101 (55)] but lack qualitative food-processing classifications, whereas others categorize levels of industrial processing [e.g., OFF (13)] but suffer from severe regional bias and missing data. Aiming to address these limitations, the Nutrition-NOVA validation benchmark was constructed as a harmonized repository that integrates structural nutritional metadata with visual evaluation assets.

Data acquisition and sparsity challenge. The benchmark is built upon two distinct repositories:

1. The Center for Food Safety Database: This database is used as the quantitative ground truth, providing highly standardized and localized dietary profiles. The extracted variables include key macronutrients (energy, protein, carbohydrate, fat, dietary fiber), critical micronutrients (calcium, iron), and precise serving-size metrics.
2. Open Food Facts Database: This crowdsourced global repository is utilized to extract qualitative NOVA food-processing classifications (Groups 1 through 4).

During the initial data integration phase, a systematic audit of the OFF repository, which comprises approximately 4.53 million commercial records, revealed a critical metadata deficit: 75.0% of the dataset (3.39 million rows) lacked a NOVA classification. This massive data sparsity, mainly attributed to missing or unparseable ingredient metadata, highlights the architectural fragility of relying solely on legacy database-only applications and mathematically justifies the necessity of the proposed VLM fallback mechanism.

#### 4.1.1 Semantic vector-space harmonization

Aiming to prevent the propagation of null values and mitigate semantic drift arising from chained fuzzy matching, a “NOVA-Strict” pre-filtering protocol was implemented. The OFF dataset was aggressively filtered to retain only the 1.13 million nodes with validated NOVA processing tiers.

Following filtration, a high-dimensional semantic harmonization process was conducted to address linguistic variations between CFS culinary names and OFF product descriptions. Both datasets were embedded into a dense vector space using a pre-trained Sentence-Transformer model (56). Cross-database linkage was then established using cosine similarity with a strict matching threshold of 0.75. This approach guaranteed that every entry in the resulting Nutrition-NOVA validation benchmark contained a high-fidelity macronutrient/serving profile (from CFS) and a validated ordinal processing tier (from OFF). Finally, 5300 food cases were filtered and used to construct the Nutrition-NOVA validation benchmark.

#### 4.1.2 Theoretical basis and mathematical justification of vector-based NOVA alignment

This semantic vector-matching framework operates under the principle of category-level processing invariance. According to standard epidemiological NOVA definitions (5, 6), processing tiers are intrinsic properties of granular food sub-categories (e.g., all unprocessed fresh fruits belong to NOVA Group 1, whereas all pure single-ingredient culinary oils belong to NOVA Group 2). To evaluate why high-dimensional vector space harmonization is necessary over conventional string matching, we mathematically contrasted Sentence-Transformer cosine similarity against traditional lexical metrics—specifically normalized Levenshtein similarity (57) and the Sørensen–Dice token coefficient (58). The comparison results of Sentence-Transformer cosine similarity against traditional lexical metrics in similar word entity but different word meaning keywords are shown in Table 3.

**Table 3:** Quantitative comparison between traditional lexical string-matching metrics and Sentence-BERT vector cosine similarity across representative Center for Food Safety (CFS) food entities and candidate Open Food Facts (OFF) commercial product strings.

CFS food entity	Candidate OFF product description	Normalized levenshtein similarity	Sørensen–dice token similarity	Sentence-BERT vector cosine similarity	SBERT decision (≥0.75)	NOVA tier & category alignment
Fresh raw apple	Raw red delicious apple	30.40%	57.10%	0.88	Accepted	NOVA 1 vs. NOVA 1 (true match: whole unprocessed fruit)
Fresh raw apple	Fresh raw apple candy	71.40%	85.70%	0.42	Rejected	NOVA 1 vs. NOVA 4 (false positive prevented: industrial candy)
Whole grain oats	Rolled whole grain oats	69.60%	85.70%	0.83	Accepted	NOVA 1 vs. NOVA 1 (true match: minimal mechanical milling)
Whole grain oats	Whole grain oats crisp bar	61.50%	75.00%	0.49	Rejected	NOVA 1 vs. NOVA 4 (false positive prevented: ultra-processed bar)
Fresh raw whole egg	Grade a large fresh egg	39.10%	44.40%	0.86	Accepted	NOVA 1 vs. NOVA 1 (true match: unprocessed animal matrix)
Fresh raw whole egg	Fresh raw whole egg powder	73.1%	88.90%	0.58	Rejected	NOVA 1 vs. NOVA 3 (false positive prevented: processed formulation)
Fresh potato	Fresh potato crispy chips	48.00%	66.70%	0.51	Rejected	NOVA 1 vs. NOVA 4 (false positive prevented: ultra-processed chips)
fresh potato	Raw unpeeled russet potato	34.60%	33.30%	0.86	Accepted	NOVA 1 vs. NOVA 1 (true match retained despite vocabulary shift)

Traditional string-matching algorithms operate strictly on character or word overlap. For instance, from Table 3, comparing a raw whole-food entity (“Fresh Raw Apple”) against an ultra-processed derivative (“Fresh Raw Apple Candy”) yields a high normalized Levenshtein similarity and a Sørensen–Dice token overlap coefficient. Because raw whole-food entity and ultra-processed derivative share 3 out of 4 word tokens (“Fresh”, “Raw”, “Apple”), traditional lexical metrics produce misleadingly high similarity scores (71.4% in Normalized Levenshtein Similarity and 85.7% in Sørensen–Dice Token Similarity) that induce false-positive alignments across divergent processing tiers. In contrast, Sentence-Transformer cosine similarity contextualizes the semantic displacement introduced by industrial modifiers like “Candy”, “Crisp Bar”, or “Powder”. As demonstrated in Table 3, Sentence-Transformer cosine similarity assigns these ultra-processed derivatives low vector similarity scores (0.42), driving them safely below our strict 0.75 filtering cutoff. Conversely, true whole-food category matches (“Fresh Raw Apple” vs. “Raw Red Delicious Apple”) sustain high vector alignment (0.88). Enforcing a strict 0.75 threshold thus restricts cross-database linkage to entries within identical culinary categories, guaranteeing that only semantically and structurally equivalent entities sharing identical NOVA classifications are harmonized into the benchmark dataset.

#### 4.1.3 Multimodal visual asset generation

Clinical VLMs operate on real-world plate imagery; therefore, the textual Nutrition-NOVA dataset was subsequently paired with a zero-shot visual evaluation. A differential web-crawling pipeline was engineered to autonomously query and download high-resolution meal images under the shared license CC-BY-4.0 corresponding to the harmonized food classes.

To ensure controlled evaluation, these visual assets were scraped specifically as isolated single-dish meal instances (e.g., a plate containing a single whole food item, a bowl of a specific culinary dish, or a distinct commercial packaged beverage) rather than complex, multi-recipe composite dining plates. This isolation ensures that benchmark evaluations measure foundational VLM visual recognition, database retrieval mapping, and qualitative NOVA classification without confounding visual noise from multi-dish occlusion, spatial boundary overlaps, or multi-recipe clutter.

Some of the food image samples corresponding to the Nutrition-NOVA validation benchmark are shown in Table 4, displaying representative visual meal instances across the four distinct NOVA processing categories from the Nutrition-NOVA validation benchmark.

**Table 4:** Sample image and their corresponding NOVA level in the benchmark.

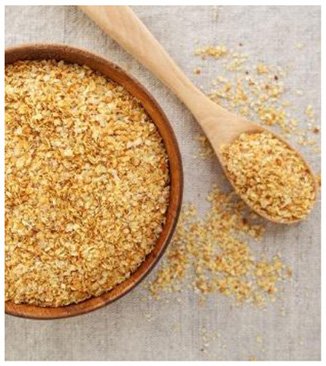	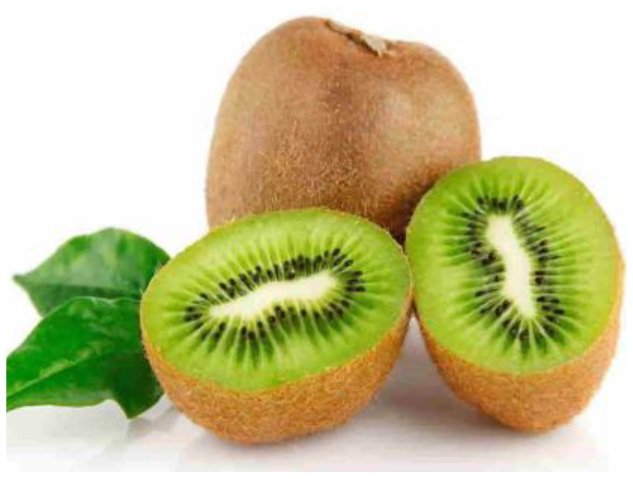	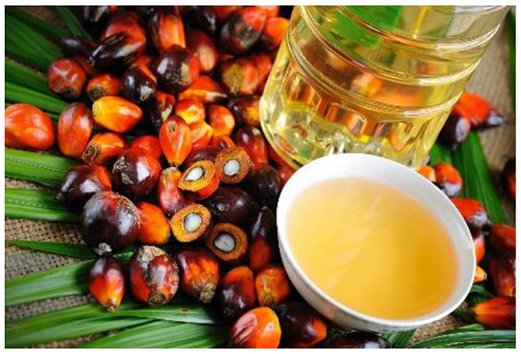
Wheat germ	Green kiwi	Palm oil
1-unprocessed or minimally processed foods	1-unprocessed or minimally processed foods	2-processed culinary ingredients
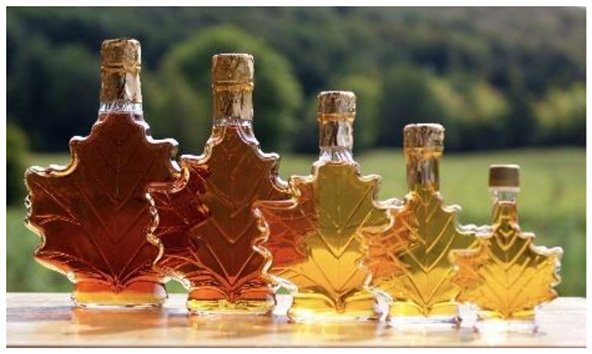	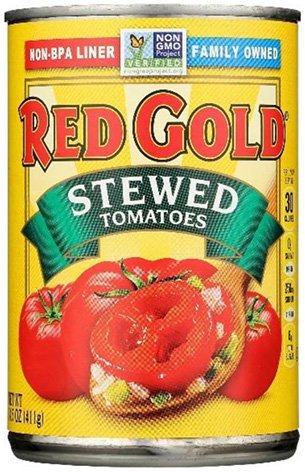	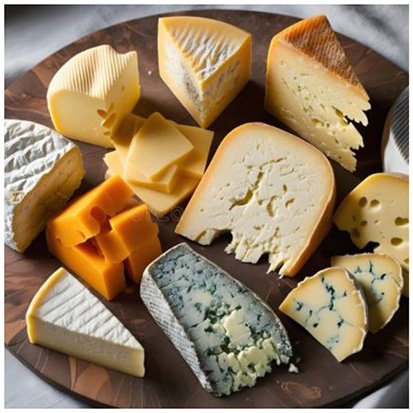
Maple syrup	Canned stewed tomato (canned food)	Cheese
2-processed culinary ingredients	3-processed foods	3-processed foods
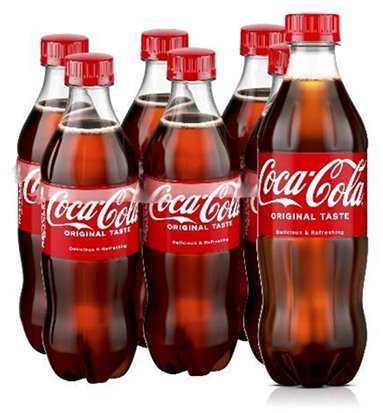	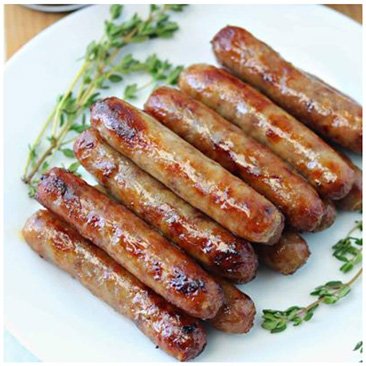	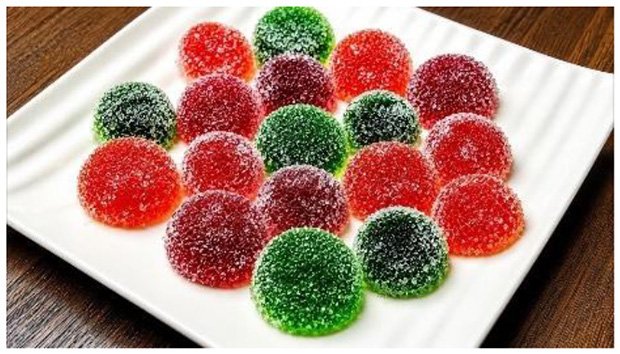
Cola	Sausage	Jelly candy
4-ultra-processed foods	4-ultra-processed foods	4-ultra-processed foods

As displayed in Table 4, the benchmark incorporates representative meal instances spanning all four NOVA processing tiers:

- **Gro**up 1 (unprocessed or minimally processed foods): This group includes raw, whole ingredients such as wheat germ and green kiwi, in which the natural food matrix remains intact without substantial industrial modification.
- Group 2 (processed culinary ingredients): This group encompasses extracted culinary bases used for cooking and seasoning, such as palm oil and pure maple syrup.
- Group 3 (processed foods): This group captures foods altered through traditional preservation or artisanal methods (e.g., salting, canning, or fermentation). Examples include canned stewed tomato and cheese.
- Group 4 (Ultra-Processed Foods): This group features complex, multi-ingredient industrial formulations containing cosmetic food additives, artificial texturizers, and chemical preservatives, such as cola, industrial sausage, and jelly candy.

Aiming to ensure varied visual representation (e.g., varying lighting, plating styles, and camera angles), exactly three images per verified food item were scraped, yielding the Nutrition-NOVA validation image repository. This isolated and cross-verified visual dataset serves as the harmonized reference benchmark for subsequent experimental evaluations, enabling a mathematically objective assessment of model performance in terms of food recognition, ordinal NOVA classification, and 2D decision matrix resolution.

#### 4.1.4 Visual complexity and multi-item plate limitations

It is important to clarify the boundary between benchmark evaluation and full real-world deployment regarding visual complexity. The Nutrition-NOVA visual repository intentionally evaluates isolated single-dish meal instances to establish controlled baseline metrics for zero-shot recognition, database mapping, and qualitative NOVA tier reasoning. While this design successfully isolates qualitative language reasoning from multi-item spatial clutter, real-world clinical dining frequently involves complex, multi-component composite meal plates (e.g., a tray containing a protein, two vegetable sides, and a commercial beverage). While Phase 1 of our system architecture incorporates region-proposal and multi-crop segmentation pipelines to parse composite scenes, full clinical validation across unconstrained, multi-dish dining environments remains a target for prospective field trials.

### 4.2 Comparative model performance and clinical metrics

Aiming to rigorously quantify the clinical decision-making assistance capabilities of the integrated MLLM framework, model predictions were evaluated across qualitative NOVA food processing levels and 2D decision matrix quadrant mappings. Evaluating ordinal classification tasks in clinical nutrition presents unique challenges. Standard accuracy metrics treat all misclassifications equally, failing to distinguish between a benign one-tier shift (e.g., classifying a NOVA 1 raw ingredient as a NOVA 2 culinary component) and a severe clinical misclassification (e.g., classifying a NOVA 4 ultra-processed food as a NOVA 1 whole food). Aiming to address this limitation, a multi-metric evaluation protocol comprising five complementary statistical metrics was established.

#### 4.2.1 Class-balanced performance via Macro-F1

Aiming to ensure that overall performance was not skewed by dataset class imbalance across the four NOVA categories, the Macro-Averaged F1-Score (*Macro-F1*) was calculated. This metric computes the unweighted mean of F1-scores across all individual processing tiers:


$$\begin{array}{l} Macro-F1=\frac{1}{C}\overset{c}{\underset{k=1}{\sum }}\frac{2*P_{k}*R_{k}}{P_{k}+R_{k}}, \end{array}$$ (2)

where C represents the distinct NOVA groups, and P_k_ and R_k_ denote the precision and recall for processing class k, respectively. Evaluating models using Macro-F1 ensures equal penalization for errors in minority categories, such as NOVA 2 culinary ingredients.

#### 4.2.2 Quantifying ordinal distance via MAE and RMSE

Aiming to measure the magnitude of prediction errors along the ordinal spectrum of food processing, the Mean Absolute Error (*MAE*) was calculated. MAE is used to measure the average linear deviation between the predicted processing level (*y*_*pred*_) and the ground-truth annotation (*y*_*gt*_):


$$\begin{array}{l} MAE=\frac{1}{N}\sum |y_{pred}-y_{gt}|. \end{array}$$ (3)

While MAE treats ordinal errors linearly, clinical risk may increase nonlinearly with error magnitude. Therefore, the root mean squared error (*RMSE*) was implemented to impose a severe quadratic penalty on large prediction deviations:


$$\begin{array}{l} RMSE=\sqrt{\frac{1}{N}\sum |y_{pred}-y_{gt}|}. \end{array}$$ (4)

An elevated RMSE relative to MAE serves as a diagnostic indicator that a model is prone to extreme clinical misclassifications.

#### 4.2.3 Clinical safety thresholds and inter-annotator agreement

Aiming to establish a practical threshold for safe point-of-care triage, adjacent accuracy (*Adj Acc* ± 1) was measured. This metric is used to calculate the percentage of predictions where the model output falls within ± 1 category of the true label, establishing the operational boundary within which automated recommendations remain clinically manageable.


$$\begin{array}{l} Adj\;Acc\pm 1=\left(\frac{1}{N}\overset{N}{\underset{i=1}{\sum }}∥\left(|y_{pred,i}-y_{gt,i}|\leq 1\right)\right), \end{array}$$ (5)

$$∥\left(\text{∗}\right)=\{\begin{array}{c} 1,\;if\;Condition\;*\;is\;True\\ 0,\;if\;Condition\;*\;is\;False \end{array}\;.$$ (6)

Rather than serving as direct proof of absolute clinical safety, adjacent accuracy quantifies computational ordinal tolerance—confirming that predictions avoid catastrophic multi-tier leaps (such as misclassifying a NOVA 4 ultra-processed food directly into a NOVA 1 whole-food tier). In real-world screening, adjacent errors carry asymmetric clinical implications: under-triage errors (NOVA level of prediction is lower than actual level) present a potential false-negative hazard by under-reporting additive exposure, whereas over-triage errors (NOVA level of prediction is higher than actual level) act as a conservative over-warning that preserves user safety. Measuring adjacent accuracy thus establishes the operational boundary for ordinal model stability, which is further contextualized via directional error analysis.

Aiming to measure the agreement between model predictions and clinical ground truth while simultaneously correcting for chance alignment, the quadratic weighted Kappa (*QWK*) was computed. By incorporating a quadratic weighting matrix, *QWK* provides a statistically robust assessment of ordinal alignment suitable for clinical validation studies.


$$\begin{array}{l} QWK=1-\frac{\sum_{i=1}^{k}\sum_{j=1}^{k}w_{ij}O_{ij}}{\sum_{i=1}^{k}\sum_{j=1}^{k}w_{ij}E_{ij}}, \end{array}$$ (7)

$$\begin{array}{l} E_{ij}=\;\frac{\left(\sum_{i=1}^{k}O_{ij}\right)*\left(\sum_{j=1}^{k}O_{ij}\right)}{N}, \end{array}$$ (8)

$$\begin{array}{l} w_{ij}=\;\frac{{\left(i-j\right)}^{2}}{{\left(k-1\right)}^{2}}. \end{array}$$ (9)

#### 4.2.4 Benchmarking protocol and web-interface sampling constraints

Aiming to guarantee a comprehensive evaluation across different computing paradigms, Table 5 details the complete model registry, categorizing the 14 evaluated LLMs and VLMs across web-based commercial interfaces, commercial snapshot endpoints, and locally hosted Hugging Face open-weight checkpoints. Open-weight models and programmatic API endpoints were evaluated across the complete harmonized dataset (*N* = 5,300). Conversely, web-based commercial LLMs (e.g., DeepSeek, ChatGPT, and Gemini web interfaces) were benchmarked on a stratified, randomized subset of *N* = 100 food items.

**Table 5:** Model registry: comparison of LLMs and checkpoint metadata.

Model	LLM category	Source of LLMs	Date of access/creation of checkpoints
Qwen (used in the platform)	Web-based commercial LLMs	https://dashscope-intl.aliyuncs.com/api	30/07/2026
DeepSeek	Web-based commercial LLMs	https://chat.deepseek.com/	28/07/2026
ChatGPT	Web-based commercial LLMs	https://chat.openai.com/	28/07/2026
Gemini	Web-based commercial LLMs	https://gemini.google.com/	28/07/2026
GPT5.4_mini	Commercial LLMs (snapshots)	University Internal Platform	28/04/2026
GPT4.1	Commercial LLMs (snapshots)	University Internal Platform	28/04/2026
Gemini 3 flash	Commercial LLMs (snapshots)	University Internal Platform	28/06/2026
Gemma3-27B	Hugging face checkpoint	Transformers library V5.3.0	04/04/2025
MedGemma-27b-it	Hugging face checkpoint	Transformers library V5.3.0	04/06/2025
Mistral-Small-3.2-24B-instruct	Hugging face checkpoint	Transformers library V5.3.0	04/08/2025
Qwen3.5-9B	Hugging face checkpoint	Transformers library V5.3.0	09/03/2026
Qwen3.6-27B	Hugging face checkpoint	Transformers library V5.3.0	22/04/2026
aya-vision-32b	Hugging face checkpoint	Transformers library V5.3.0	04/04/2025
LFM2-VL-3B	Hugging face checkpoint	Transformers library V5.3.0	26/11/2025

This sampling protocol is necessary due to operational constraints inherent set by graphical web frontends, which often lack automated batch-processing endpoints and enforce aggressive request-rate limits, anti-bot verification measures, and session timeouts. A randomized sample size of *N* = 100 provides a statistically robust evaluation scale while preserving the original NOVA class distribution and maintaining the integrity of the zero-shot evaluation under web-interface execution limits.

Aiming to maintain strict experimental reproducibility across all evaluated models, Figure 7 outlines the exact system prompt architectures engineered for the proposed multimodal framework:

- Panel (a) — End-to-end visual prompt schema: Ingests raw meal plate images and performs simultaneous visual food recognition, ordinal NOVA classification, and 2D clinical decision-matrix quadrant mapping within a single inference pass.
- Panel (b) — Decoupled text prompt schema: Receives standardized food-item text strings and implements qualitative ingredient auditing, keyword-based trigger scanning (detecting industrial emulsifiers and cosmetic additives), and generation of the corresponding clinical rationale.

**Figure 7:**
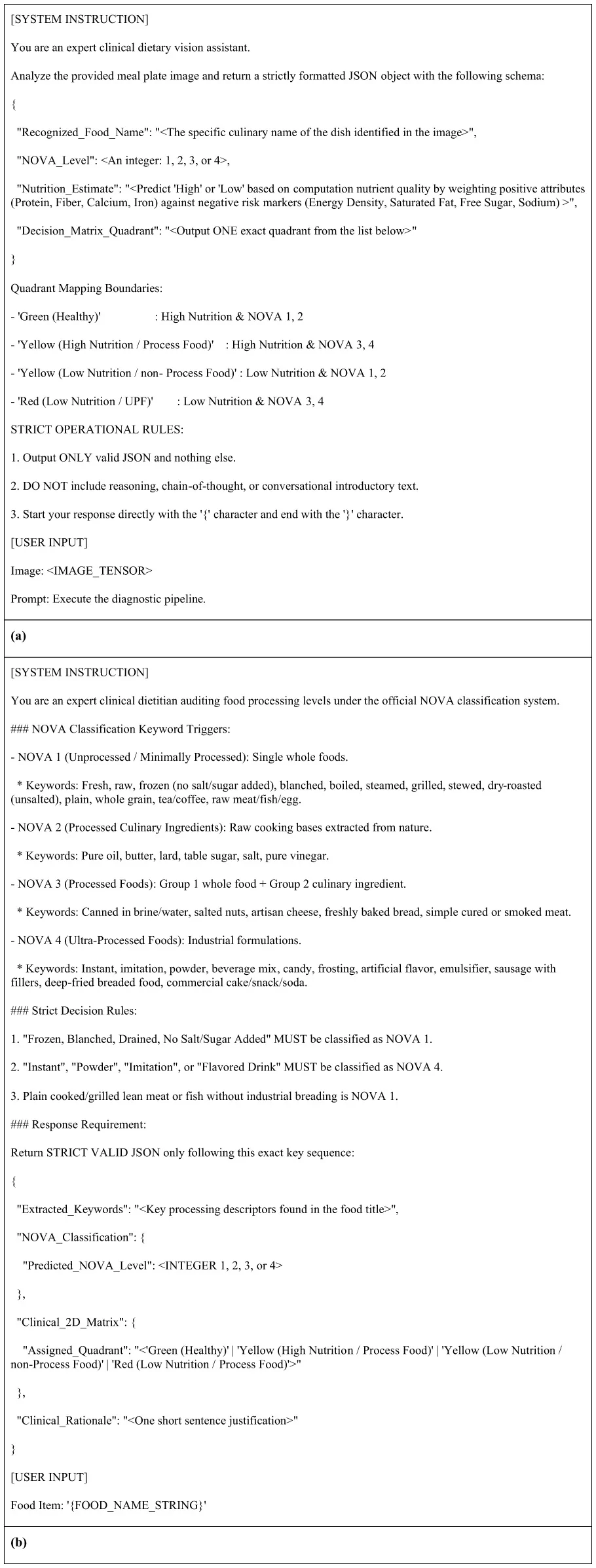
Prompt templates for the MLLM dietary framework. **(a)** Prompt for end-to-end visual food recognition, NOVA classification, and 2D decision matrix mapping. **(b)** Prompt for decoupled text-based semantic processing auditing and clinical rationale generation.

#### 4.2.5 Scope of clinical decision support and non-diagnostic disclaimer

It is important to emphasize that the proposed 2D decision matrix functions as an automated dietary auditing and risk-screening utility designed for personal health monitoring and clinical decision-support. The platform does not perform direct medical diagnosis or predict individual oncological outcomes. While ultra-processed food consumption is extensively linked to chronic metabolic disorders and elevated cancer incidence in large-scale epidemiological cohorts (5, 6), translating computational meal screening into individual clinical risk prognosis assistance requires prospective clinical validation trials. Future deployment in healthcare settings will position this framework as an adjunct screening tool to assist dietitians and users in identifying ultra-processed food exposure rather than an autonomous diagnostic device.

### 4.3 End-to-end vision-language model visual benchmark

Aiming to evaluate the feasibility of real-time visual meal auditing at the point of care, an end-to-end multimodal benchmark was used to assess direct image-to-verdict prediction. The VLMs received raw meal plate images from the Nutrition-NOVA validation benchmark and were tasked with simultaneously performing dish identification, qualitative processing inference, and structured metric generation without intermediate textual prompts. Table 6 presents the evaluation results of the VLM benchmark, including quantitative performance metrics for VLMS performing direct visual food recognition and NOVA classification.

**Table 6:** Quantitative performance evaluation of vision-language models on end-to-end visual food recognition and NOVA processing classification.

Model	Macro F1	MAE	RMSE	Adj Acc	QWK
Qwen API framework (100 random samples)					
Qwen-VL-max (used in the platform)	0.457	0.825	1.316	77.5	0.439
Qwen API framework (all samples)					
Qwen (used in the platform)	0.486	0.811	1.308	77.7	0.467
Web-based commercial LLMs (most recently updated) (100 random samples)					
DeepSeek	0.452	0.780	1.305	76.0	0.468
ChatGPT	0.285	1.580	2.020	49.0	0.078
Gemini	0.412	0.830	1.345	75.0	0.428
Commercial LLMs (snapshots) (100 random samples)					
GPT5.4_mini	0.518	0.710	1.265	77.0	0.492
GPT4.1	0.552	0.600	1.115	83.0	0.588
Gemini 3 flash	0.315	1.410	1.940	53.0	0.142
Hugging face checkpoint inference (all samples)					
Mistral-small-3.2-24B-instruct	0.311	1.111	1.549	64.7	0.352
Qwen3.5-9B	0.240	1.149	1.496	69.6	0.316
Qwen3.6-27B	0.318	1.115	1.450	71.2	0.345
Gemma3-27B	0.297	0.958	1.398	66.7	0.367
MedGemma-27b-it	0.307	1.046	1.479	69.9	0.346
aya-vision-32b	0.311	1.027	1.489	71.3	0.381
LFM2-VL-3B	0.298	1.097	1.522	65.8	0.366

#### 4.3.1 Multimodal dish recognition and spatial perception

The empirical visual benchmark demonstrates that modern vision-language architectures possess strong capabilities for direct plate recognition. As shown in Table 6, among the 100 randomly selected test samples, commercial snapshot endpoints achieved the strongest overall visual performance. GPT 4.1 achieved the highest overall benchmark score (*Macro-F1* = 0.552, *MAE* = 0.600, *Adj Acc* ± 1 = 83.0%, *QWK* = 0.588), closely followed by GPT5.4_mini (*Macro-F1* = 0.518, *MAE* = 0.710, *Adj Acc* ± 1 = 77.0%, *QWK* = 0.492). Among the web-based commercial interfaces evaluated on the same 100 randomly selected samples, DeepSeek (*Macro-F1* = 0.452, *MAE* = 0.780, *QWK* = 0.468) and Gemini (*Macro-F1* = 0.412, *MAE* = 0.830) substantially outperformed ChatGPT (*Macro-F1* = 0.285, *MAE* = 1.580, *QWK* = 0.078). Among locally hosted open-weight models executed on HPC compute nodes, Qwen3.6-27B achieved the strongest performance (*Macro-F1* = 0.318, *MAE* = 1.115, *QWK* = 0.345), outperforming specialized domain architectures such as Aya-Vision-32b (*Macro-F1* = 0.311), Mistral-Small-3.2-24B-Instruct (*Macro-F1* = 0.311), MedGemma-27b-it (*Macro-F1* = 0.307), and Gemma3-27B (*Macro-F1* = 0.297).

#### 4.3.2 Experimental instantiation vs. framework agnosticism and dataset scale stability

Notably, the deployment of Qwen-VL-Max within the online platform serves purely as a practical and representative baseline for experimental benchmarking. As shown in Table 6, across the full dataset benchmark (*N* = 5,300), the proposed framework instantiated with Qwen-VL-Max achieved robust performance (*Macro-F1* = 0.486, *MAE* = 0.811, *Adj Acc* ± 1 = 77.7%, and *QWK* = 0.467). Crucially, evaluation on the 100-sample subset yielded nearly identical results (*Macro-F1* = 0.457, *MAE* = 0.825, *Adj Acc* ± 1 = 77.5%, and *QWK* = 0.439).

The small performance variance observed across evaluation scales (**Δ***Adj Acc* ± 1 = 0.2%, **Δ***Macro-F1* = 0.029) demonstrates two key insights:

- Framework Statistical Stability: The overall multimodal pipeline architecture exhibits high consistency and robustness without performance degradation or volatility when scaled from the 100-sample subset to thousands of samples.
- Benchmark Subset Validity: The 100-sample randomized subset serves as an empirically sound and representative dataset, validating its use for comparative benchmarking against web-based commercial models constrained by rate limits.

Furthermore, the framework architecture is inherently model-agnostic; therefore, any higher-performing VLM backends can be directly integrated into the vision module without altering the underlying pipeline architecture, database retrieval layer, or prompt-orchestration schema.

#### 4.3.3 Clinical safety boundaries and adjacent alignment

Despite the challenges associated with visual perception, top-performing vision models maintained strong clinical safety margins. GPT4.1, Qwen-VL-Max, GPT5.4_mini, and DeepSeek achieved high adjacent accuracy scores of 83.0%, 77.7%, 77.0%, and 76.0%, respectively. These high adjacent-alignment scores confirm that when visual models misclassify an item, their errors are almost exclusively restricted to adjacent processing tiers (such as confusing NOVA 1 minimally processed foods with NOVA 2 culinary ingredients or NOVA 3 processed foods), rarely producing extreme misclassifications that would compromise clinical safety.

### 4.4 Decoupled text-only LLM benchmark on NOVA classification and framework modularity

A decoupled text-only evaluation on NOVA classification was conducted to assess the internal nutritional reasoning engine independently of visual perception errors. Uncorrupted culinary food names from the harmonized dataset were provided directly to various open-weight and commercial LLM backends to isolate their capacity to reason about industrial processing signatures, ingredient matrices, and qualitative NOVA boundaries. The results of the text-based LLM evaluation for NOVA classification are presented in Table 7, which summarizes the benchmarking results of decoupled text-based LLMs using standardized food item names.

**Table 7:** Quantitative performance evaluation of decoupled large language models on semantic NOVA processing classification given standardized food names.

Model	Macro F1	MAE	RMSE	Adj Acc	QWK
Qwen API (100 random samples)					
Qwen-max (used in the platform)	0.557	0.760	1.311	78.0	0.494
Qwen API (all samples)					
Qwen-max (used in the platform)	0.477	0.875	1.418	73.1	0.404
Web-based commercial LLMs (most recently updated) (100 random samples)					
DeepSeek	0.537	0.71	1.277	78.0	0.518
ChatGPT	0.272	1.65	2.095	47.0	0.044
Gemini	0.446	0.76	1.327	78.0	0.483
Commercial LLMs (snapshots) (100 random samples)					
GPT5.4_mini	0.610	0.64	1.233	80.0	0.555
GPT4.1	0.631	0.50	1.000	87.0	0.675
Gemini 3 Flash	0.298	1.49	2.007	51.0	0.114
Hugging face checkpoint inference (all samples)					
Mistral-small-3.2-24B-Instruct	0.497	0.953	1.507	69.9	0.372
Qwen3.5-9B	0.248	1.554	2.066	42.0	0.057
Qwen3.6-27B	0.306	1.323	1.769	54.8	0.179
MedGemma-27b-it	0.269	1.331	1.876	54.0	0.247
Gemma3-27B	0.269	1.277	1.704	56.6	0.258

#### 4.4.1 Semantic processing awareness and qualitative reasoning

The text-only benchmark revealed clear performance variations across model architectures and execution interfaces. As shown in Table 7, commercial snapshot models achieved the strongest reasoning performance, with GPT4.1 leading the benchmark (*Macro-F1* = 0.631, *MAE*= 0.500, *Adj Acc* ± 1 = 87.0%, *QWK* = 0.675), closely followed by GPT5.4_mini (*Macro-F1* = 0.610, *MAE* = 0.640, *Adj Acc* ± 1 = 80.0%, *QWK* = 0.555). Structurally constrained API endpoints consistently outperformed unconstrained web-based interfaces, which frequently generated conversational fluff or violated JSON schema constraints (e.g., ChatGPT web base recording *Macro-F1* = 0.272, *MAE* = 1.650, *QWK* = 0.044). Among the open-weight checkpoints evaluated across the complete dataset, Mistral-Small-3.2-24B-Instruct demonstrated the strongest alignment in text-based reasoning (*Macro-F1* = 0.497, *MAE* = 0.953, *Adj Acc* ± 1 = 69.9%), outperforming larger general-purpose open models, including Qwen3.6-27B (*Macro-F1* = 0.306), Gemma3-27B (*Macro-F1* = 0.269), and MedGemma-27b-it (*Macro-F1* = 0.269).

#### 4.4.2 Experimental instantiation vs. scale stability

As the sample experimental setting, Qwen-Max was deployed in the platform as an accessible baseline model for experimental validation. As shown in Table 7, when evaluated on 100 randomly selected samples, Qwen-Max achieved *Macro-F1* = 0.557, *MAE* = 0.760, *Adj Acc* ± 1 = 78.0%, and *QWK* = 0.494. When scaled across the full harmonized dataset (*N* = 5,300), its performance remained relatively stable (*Macro-F1* = 0.477, *MAE* = 0.875, *Adj Acc* ± 1 = 73.1%, and *QWK* = 0.404). This narrow delta between sample scales confirms the statistical reliability of the 100-sample evaluation proxy for rate-limited web-accessible models and demonstrates the operational stability of the prompting architecture across thousands of concurrent inputs.

#### 4.4.3 Plug-and-play architecture and backend agnosticism

A core contribution of this work lies in the modular and backend-agnostic architecture of the proposed framework. The selection of Qwen-Max and Qwen-VL-Max for platform implementation represents an experimental baseline choice. The database retrieval layer standardizes food names and nutritional information into structured JSON schemas prior to reasoning, thereby allowing the framework to function as a plug-and-play system.

If a healthcare institution requires maximum predictive precision, then higher-performing engines such as GPT4.1 or GPT5.4_mini can be immediately substituted into the framework. Conversely, if on-premise data privacy and compliance are required, open-source checkpoints (such as Mistral-Small-3.2-24B) can be deployed on local HPC clusters without modifying the prompt templates, database linkages, or clinical decision-matrix mapping logic.

#### 4.4.4 Cross-modal reasoning stability

Comparing the text-only benchmark with the end-to-end VLM benchmark in Section 4.3 illustrates the overall cross-modal stability and architectural advantages of the multi-phase design. For the platform baseline (Qwen-VL-Max and Qwen-Max), the full end-to-end visual framework achieved an adjacent accuracy (*Adj Acc* ± 1 = 73.1%) outperforming the decoupled text-only baseline (*Adj Acc* ± 1 = 77.7%). This finding indicates that, rather than introducing perceptual degradation, the integration of visual recognition with structured knowledge retrieval yields superior and more consistent clinical alignment compared with processing standalone textual culinary names.

This performance advantage stems from the systemic synergy across of the proposed four-phase pipeline, where Phase 1 visual feature extraction and Phase 2 knowledge graph retrieval actively enrich the diagnostic context before qualitative LLM reasoning. Specifically, Phase 1 captures implicit visual indicators of food preparation, such as deep-fried breading, visible surface oil sheens, and industrially formulated sauce matrices, which are frequently omitted or remain ambiguous in simple text descriptions. Concurrently, the intermediate database retrieval layer standardizes open-vocabulary dish names into canonical entities with verified nutritional profiles, effectively reducing the impact of visual noise and semantic ambiguity on the subsequent reasoning process. Consequently, the full visual pipeline provides the qualitative LLM reasoning with enriched multimodal context, proving that the proposed architecture successfully converts raw visual inputs into highly reliable, clinically grounded diagnostic intelligence.

### 4.5 System latency and resource efficiency

Deploying automated dietary monitoring frameworks in real-time clinical point-of-care or mobile edge environments requires balancing predictive accuracy with end-to-end execution latency, token payload overhead, and operational deployment costs. Table 8 outlines the step-wise execution latency and token payload consumption across commercial cloud APIs, web platforms, and local on-premise GPU clusters over five evaluation trials.

**Table 8:** Computational efficiency: step-wise latency and token consumption. All the values are presented in mean ± std in five trials.

Test image: 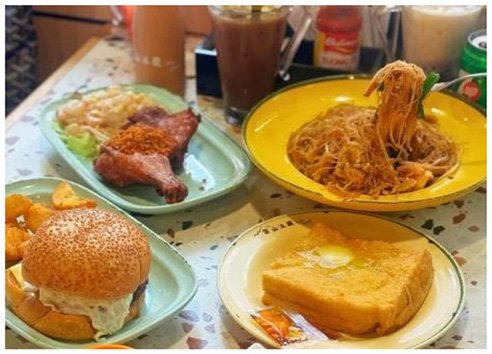	Time (mean ±STD, s)	Input token (mean ±STD)	Output token (mean ±STD)
Qwen (used in the proposed framework in experiments)			
Step 1: dish recognition	5.85 ± 2.10	1,378 ± 1	88 ± 3
Step 2: nutrition retrieval (from CFS database)	1.24 ± 0.22	/	/
Step 3: NOVA classification	2.81 ± 1.41	312 ± 2	1,873 ± 47
Total	9.90 ± 1.25	1,690 ± 1	1,961 ± 51
Web-based online platform (Gemini)			
Step 1: dish recognition	/	/	/
Step 2: nutrition retrieval	/	/	/
Step 3: NOVA classification	/	/	/
Total **(no statistics for each step)**	10.14 ± 1.25	1,684 ± 101	2,893 ± 46
Hugging face checkpoint (Gemma3-27B)			
Step 1: dish recognition	21.15 ± 0.85	328 ± 1	195 ± 2
Step 2: nutrition retrieval	1.28 ± 0.31	/	/
Step 3: NOVA classification	14.50 ± 0.35	512 ± 0	148 ± 1
Total	36.93 ± 0.50	840 ± 1	343 ± 1

#### 4.5.1 Phase-wise latency profiling across deployment paradigms

System response times were profiled across local GPU hardware and commercial API endpoints over five evaluation trials to determine real-time deployment feasibility.

- Deployed Platform Backend (Qwen API): Based on the running time shown in Table 8, the multi-tier pipeline achieved a highly responsive total latency of 9.90 ± 1.25 s. Phase 1 (multi-stage food recognition) consumed 5.85 ± 2.10 s, representing the visual feature extraction overhead. Phase 2 (dual-path nutrition label profiling) was executed in near real-time at 1.24 ± 0.22 s, demonstrating the efficiency of structured database indexing. Phases 3 and 4 (automated NOVA processing classification, unified 2D decision matrix, and risk quadrants) were completed in 2.81 ± 1.41 s.
- Web-Based Commercial Platform (Gemini Web UI): Based on the running time presented in Table 8, the unified end-to-end execution was completed in 10.14 ± 1.25 s, exhibiting processing speeds nearly identical to the proposed platform API baseline. However, this end-to-end prediction lacks step-wise visibility.
- Local On-Premise Execution (Gemma3-27B HPC Checkpoint): Based on the running time shown in Table 8, local execution on 2^*^ NVIDIA H20 (40 GB VRAM) cluster recorded a total pipeline latency of 36.93 ± 0.50 s. Step 1 visual embedding required 21.15 ± 0.85 s, while Step 3 autoregressive text generation required 14.50 ± 0.35 s. Local inference introduces higher latency compared with web-based interface due to limited computational resources but provides an isolated, zero-egress environment that is essential for privacy-sensitive hospital deployments.1

#### 4.5.2 Token overhead dynamics and output payload control

Token consumption was audited across all inference runs to evaluate operational efficiency for large-scale population health monitoring.

- Deployed Platform Backend (Qwen API): Based on the token cost shown in Table 8, Phase 1 visual patch processing consumed 1,378 ± 1 input tokens, yielding a concise 88 ± 3 output token dish descriptor. In Phase 3, system prompts containing explicit NOVA boundary rules and keyword triggers required 312 ± 2 input tokens and generated 1,873 ± 47 output tokens, delivering full clinical rationales and matrix mappings. Across the entire pipeline, total token consumption averaged 1,690 ± 1 input tokens and 1,961 ± 51 output tokens.
- Web-Based Commercial Platform (Gemini Web UI): Based on the token cost presented in Table 8, single-turn execution required 1,684 ± 101 input tokens and generated 2,893 ± 46 output tokens due to unconstrained conversational responses.
- Local On-Premise Execution (Gemma3-27B HPC Checkpoint): Based on the token cost shown in Table 8, strict JSON schema constraints restricted the total output tokens to 343 ± 1 (195 ± 2 tokens in Phase 1 and 148 ± 1 tokens in Phase 3), dramatically reducing local memory bandwidth utilization and accelerating output decoding speed.

## 5 Conclusion

This work presented a decoupled multimodal and IoT-enabled dietary decision framework designed to address the inherent limitations of legacy database-only applications and end-to-end VLMs. The diagnostic workflow is structured into four distinct phases: visual food recognition via VLMs, standardized nutrient mapping via knowledge graph database retrieval, qualitative processing classification using LLMs, and 2D decision-matrix analysis. This architecture successfully isolates perceptual visual ambiguity from clinical knowledge reasoning. Furthermore, to address severe metadata sparsity in existing crowdsourced repositories, where 75.0% of OFF records lacked explicit processing tiers, the Nutrition-NOVA Validation Benchmark was constructed through dense vector-space semantic harmonization, establishing a high-fidelity multimodal ground truth (N = 5300) for automated point-of-care dietary auditing.

Comprehensive empirical evaluation demonstrated that the proposed architecture achieves robust and clinically safe predictions across qualitative NOVA processing tiers and 2D clinical decision-matrix quadrant mappings. Top-performing VLM implementations achieved adjacent accuracy scores ranging from 75.0% to 83.0%, confirming that prediction errors generally remained within adjacent processing tiers without producing extreme, potentially clinically dangerous misclassifications. Benchmark validation further revealed strong cross-modal performance retention between direct visual plate evaluation (*Adj Acc* ± 1 = 77.7%) and text-only upper bounds (*Adj Acc* ± 1 = 73.1%). This minimal performance gap proves that visual recognition errors introduce negligible downstream degradation, confirming the reliability of the overall framework for point-of-care dietary assessment.

The experiments confirmed the operational flexibility and deployability of the proposed framework. Enforcing a standardized JSON schema across intermediate data layers allows for complete plug-and-play backend replaceability. While Qwen-VL-Max and Qwen-Max were deployed as experimental baseline prototypes for platform benchmarking, healthcare systems can seamlessly interchange high-accuracy commercial cloud APIs (such as GPT4.1 or DeepSeek) with private, open-weight models executed on local edge or HPC infrastructure. Profiling inference latency and token consumption highlighted distinct operational tradeoffs. Commercial platform endpoints achieved responsive execution (9.90 ± 1.25 s total latency) suitable for interactive meal scanning, while leveraging asynchronous batch processing reduced operational API execution costs by 50% for cohort-scale population health monitoring.

The practical utility of the proposed framework lies in the establishment of a seamless collaborative loop connecting patients or caregivers, the framework, and care teams/experts. Aiming to demonstrate the practical clinical utility and real-world deployability of the proposed platform, Figure 8 illustrates the translational collaborative workflow connecting patients/caregivers, the MLLM framework, and clinical care teams.

**Figure 8:**
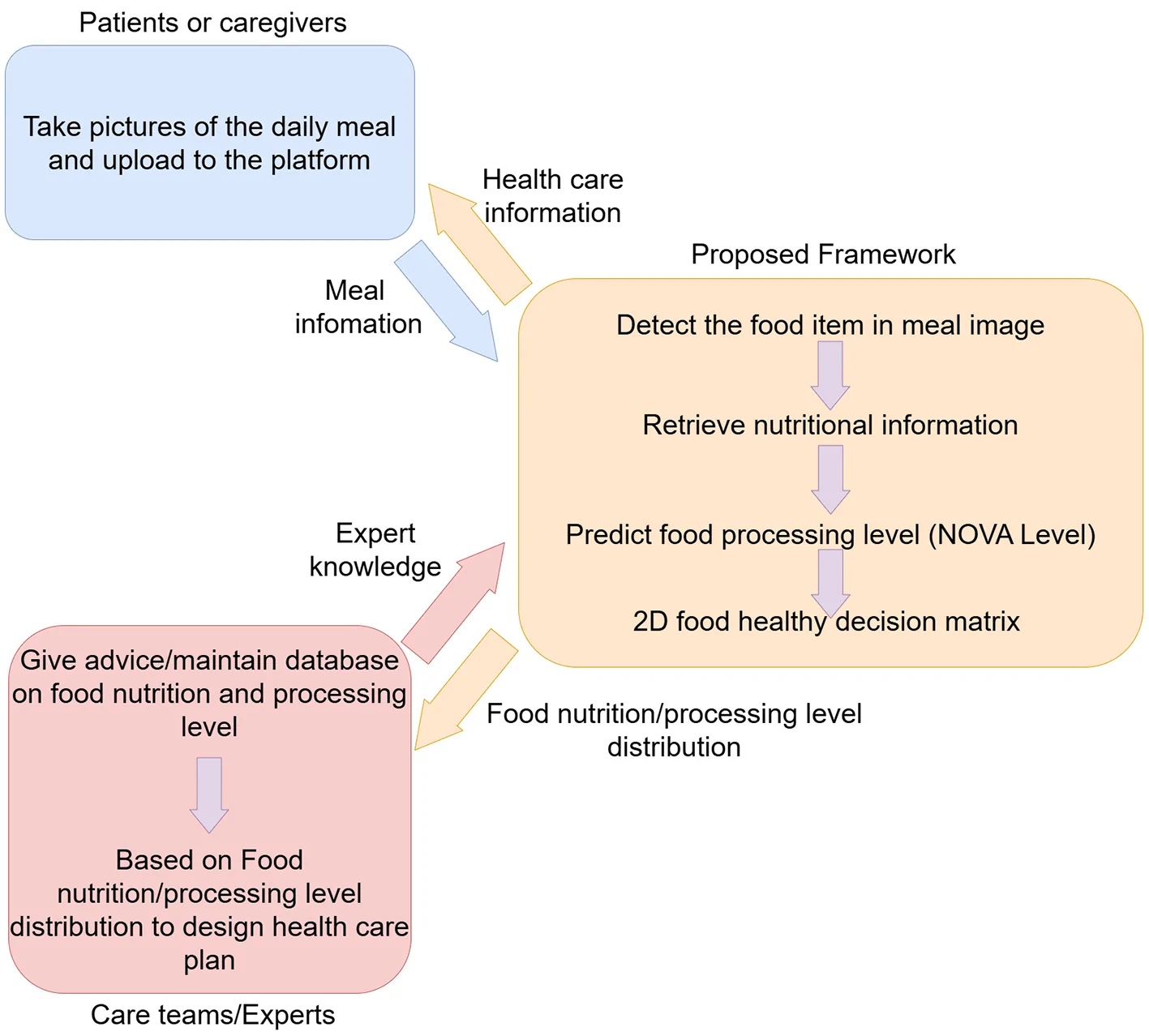
Potential collaborative workflow connecting patients, the proposed framework, and clinical care teams.

As detailed in the ecosystem architecture in Figure 8, the collaborative information flow operates across three interconnected nodes:

- Patient and caregiver interface (blue box): In daily domestic routines, patients or caregivers photograph their daily meals and upload the images to the platform as meal information. The framework processes the images and immediately returns personalized healthcare information back to the user, providing instant point-of-care feedback and behavioral guidance without requiring manual nutritional logging.
- Core Framework Engine (orange box): The platform executes an internal four-stage diagnostic pipeline on the uploaded meal imagery:

1. Detection of the food item in meal image: This stage involves regional segmentation and zero-shot VLM recognition.
2. Retrieval of nutrition information: This stage obtains 100 g-normalized macronutrient and micronutrient vectors from databases.
3. Prediction of food-processing level (NOVA Level): This stage audits ingredient matrices and chemical additive markers to assign food items to NOVA Groups 1–4.
4. 2D food healthy decision matrix: This stage maps dual-axis outputs into the 2D decision matrix to establish clear diagnostic verdicts (green, yellow, or red).

- Care Teams and Domain Experts Interface (red box): Clinical care teams and domain experts actively contribute to the ecosystem by supplying expert knowledge, maintaining localized food and nutrition databases, and refining food-processing audit rules (give advice/maintain database on food nutrition and processing level). In return, the framework aggregates individual meal audits to generate longitudinal reports on patients' dietary intake, nutritional profiles, and food-processing level distributions, allowing clinicians to monitor dietary patterns.

Based on these system-level processing distributions, care teams can evaluate a patient's overall exposure to UPFs, design targeted medical nutrition therapy regimens, and deliver personalized clinical guidance directly to patients or caregivers, thereby establishing a closed-loop digital health ecosystem.

Future work will focus on expanding the underlying knowledge graph to incorporate a broader array of global regional cuisines and specialized medical nutrition therapy protocols, such as renal and diabetic dietary restrictions. Crucially, to transition from offline benchmark screening to prospective clinical deployment, future iterations will replace symmetric evaluation metrics with asymmetric, risk-weighted clinical loss functions that heavily penalize under-triage misclassifications over conservative over-warning. Additionally, real-time mobile deployment will be integrated into prospective clinical trials to evaluate the real-world impact of the framework on long-term dietary compliance, automated metabolic risk monitoring, and clinical workflow optimization.

## Data Availability

The data analyzed in this study will be made available by the corresponding author upon request.

## References

[1] Willett W Rockström J Loken B Springmann M Lang T Vermeulen S . Food in the anthropocene: the EAT–Lancet commission on healthy diets from sustainable food systems. Lancet. (2019) 393:447–92. doi: 10.1016/S0140-6736(18)31788-4 30660336

[2] Mozaffarian D. Dietary and policy priorities for cardiovascular disease, diabetes, and obesity: a comprehensive review. Circulation. (2016) 133:187–225. doi: 10.1161/CIRCULATIONAHA.115.018585 26746178 PMC4814348

[3] Ordovas JM Ferguson LR Tai ES Mathers JC. Personalised nutrition and health. Br Med J. (2018) 361:k2173. doi: 10.1136/bmj.k2173 29898881 PMC6081996

[4] Cordova R Viallon V Fontvieille E Peruchet-Noray L Jansana A Wagner KH . Consumption of ultra-processed foods and risk of multimorbidity of cancer and cardiometabolic diseases: a multinational cohort study. Lancet Reg Health Eur. (2023) 35:100771. doi: 10.1016/j.lanepe.2023.100771 38115963 PMC10730313

[5] Monteiro CA Cannon G Moubarac J-C Levy RB Louzada MLC Jaime PC. The UN decade of nutrition, the NOVA food classification and the trouble with ultra-processing. Public Health Nutr. (2018) 21:5–17. doi: 10.1017/S1368980017000234 28322183 PMC10261019

[6] Monteiro CA Cannon G Levy RB Moubarac JC Louzada ML Rauber F . Ultra-processed foods: what they are and how to identify them. Public Health Nutr. (2019) 22:936–41. doi: 10.1017/S1368980018003762 30744710 PMC10260459

[7] Abeltino A Riente A Bianchetti G Serantoni C De Spirito M Capezzone S . Digital applications for diet monitoring, planning, and precision nutrition for citizens and professionals: a state of the art. Nutr Rev. (2025) 83:e574–601. doi: 10.1093/nutrit/nuae035 38722240 PMC11986332

[8] Yan R Luo H Lu J Liu D Posluszny H Dhaliwal MP . DietAI24 as a framework for comprehensive nutrition estimation using multimodal large language models. Commun Med. (2025) 5:458. doi: 10.1038/s43856-025-01159-0 41193610 PMC12589391

[9] Menichetti G Ravandi B Mozaffarian D Barabási AL. Machine learning prediction of the degree of food processing. Nat Commun. (2023) 14:2312. doi: 10.1038/s41467-023-37457-1 37085506 PMC10121643

[10] Konstantakopoulos FS Georga EI Fotiadis DI. A review of image-based food recognition and volume estimation artificial intelligence systems. IEEE Rev Biomed Eng. (2024) 17:136–52. doi: 10.1109/RBME.2023.3283149 37276096

[11] Lucassen DA Willemsen RF Geelen A Brouwer-Brolsma EM Feskens EJ. The accuracy of portion size estimation using food images and textual descriptions of portion sizes: an evaluation study. J Hum Nutr Diet. (2021) 34:945–52. doi: 10.1111/jhn.12878 33761165 PMC9291996

[12] Campbell JL Schofield G Tiedt HR Zinn C. Artificial intelligence applications for assessing ultra-processed food consumption: a scoping review. Br J Nutr. (2026) 135:463–73. doi: 10.1017/S000711452510593X 41423345 PMC12929014

[13] Open Food Facts. Open food facts: world food products database. (2026). Available online at: https://world.openfoodfacts.org/ (Accessed July 9, 2026).

[14] Shim JS Oh K Kim HC. Dietary assessment methods in epidemiologic studies. Epidemiol Health. (2014) 36:e2014009. doi: 10.4178/epih/e2014009 25078382 PMC4154347

[15] Ma Y Olendzki BC Pagoto SL Hurley TG Magner RP Ockene IS . Number of 24-hour diet recalls needed to estimate energy intake. Ann Epidemiol. (2009) 19:553–9. doi: 10.1016/j.annepidem.2009.04.010 19576535 PMC2803049

[16] Shahar D Shai I Vardi H Brener-Azrad A Fraser D. Development of a semi-quantitative food frequency questionnaire (FFQ) to assess dietary intake of multiethnic populations. Eur J Epidemiol. (2003) 18:855–61. doi: 10.1023/A:1025634020718 14561044

[17] Sharma S. Development and use of FFQ among adults in diverse settings across the globe. Proc Nutr Soc. (2011) 70:232–51. doi: 10.1017/S0029665110004775 21284909

[18] Ravelli MN Schoeller DA. Traditional self-reported dietary instruments are prone to inaccuracies and new approaches are needed. Front Nutr. (2020) 7:90. doi: 10.3389/fnut.2020.00090 32719809 PMC7350526

[19] Zuniga K McAuley E. Considerations in selection of diet assessment methods for examining the effect of nutrition on cognition. J Nutr Health Aging. (2015) 19:333–40. doi: 10.1007/s12603-014-0566-5 25732219 PMC5800305

[20] Vandelanotte C Müller AM Short CE Hingle M Nathan N Williams SL . Past, present, and future of eHealth and mHealth research to improve physical activity and dietary behaviors. J Nutr Educ Behav. (2016) 48:219–28. doi: 10.1016/j.jneb.2015.12.006 26965100

[21] Fiordelli M Diviani N Schulz PJ. Mapping mHealth research: a decade of evolution. J Med Internet Res. (2013) 15:e2430. doi: 10.2196/jmir.2430 23697600 PMC3668610

[22] Fukagawa NK McKillop K Pehrsson PR Moshfegh A Harnly J Finley J. USDA's FoodData central: what is it and why is it needed today? Am J Clin Nutr. (2022) 115:619–24. doi: 10.1093/ajcn/nqab397 34893796

[23] Carter MC Burley VJ Nykjaer C Cade JE. Adherence to a smartphone application for weight loss compared to website and paper diary: pilot randomized controlled trial. J Med Internet Res. (2013) 15:e32. doi: 10.2196/jmir.2283 23587561 PMC3636323

[24] Darby A Strum MW Holmes E Gatwood J. A review of nutritional tracking mobile applications for diabetes patient use. Diabetes Technol Ther. (2016) 18:200–12. doi: 10.1089/dia.2015.0299 26950679

[25] Wang W Min W Li T Dong X Li H Jiang S. A review on vision-based analysis for automatic dietary assessment. Trends Food Sci Technol. (2022) 122:223–37. doi: 10.1016/j.tifs.2022.02.017

[26] Liu C Cao Y Luo Y Chen G Vokkarane V Ma Y. “Deepfood: deep learning-based food image recognition for computer-aided dietary assessment,” in *International Conference on Smart Homes and Health Telematics.* Cham: Springer (2016) 37–48. doi: 10.1007/978-3-319-39601-9_4

[27] Wang H Tian H Ju R Ma L Yang L Chen J . Nutritional composition analysis in food images: an innovative Swin Transformer approach. Front Nutr. (2024) 11:1454466. doi: 10.3389/fnut.2024.1454466 39469326 PMC11514735

[28] Thames Q Karpur A Norris W Xia F Panait L Weyand T . “Nutrition5k: towards automatic nutritional understanding of generic food,” in *2021 IEEE/CVF Conference on Computer Vision and Pattern Recognition.* Nashville, TN: IEEE (2021). 8899–8907. doi: 10.1109/CVPR46437.2021.00879

[29] Kwan Z Zhang W Wang Z Ng AB See S. Nutrition estimation for dietary management: a transformer approach with depth sensing. IEEE Trans Multim. (2025) 27:6047–58. doi: 10.1109/TMM.2025.3565966

[30] Sempionatto JR Montiel VRV Vargas E Teymourian H Wang J. Wearable and mobile sensors for personalized nutrition. ACS Sens. (2021) 6:1745–60. doi: 10.1021/acssensors.1c00553 34008960

[31] Shi Z Li X Shuai Y Lu Y Liu Q. The development of wearable technologies and their potential for measuring nutrient intake: towards precision nutrition. Nutr Bull. (2022) 47:388–406. doi: 10.1111/nbu.12581 36134894

[32] Han Y Cheng Q Wu W Huang Z. DPF-Nutrition: food nutrition estimation via depth prediction and fusion. Foods. (2023) 12:4293. doi: 10.3390/foods12234293 38231726 PMC10706621

[33] Makhsous S Bharadwaj M Atkinson BE Novosselov IV Mamishev AV. DietSensor: automatic dietary intake measurement using mobile 3D scanning sensor for diabetic patients. Sensors. (2020) 20:3380. doi: 10.3390/s20123380 32549356 PMC7349497

[34] Agrawal K Kumar N Singh A Lu C Leung MF. Artificial intelligence in food and nutrition science: a paradigm-centric review of computational frameworks and system-level integration. Food Funct. (2026) 17:7908–41. doi: 10.1039/D6FO02990F 42610782

[35] Agrawal K Goktas P Kumar N Leung M-F. Artificial intelligence in personalized nutrition and food manufacturing: a comprehensive review of methods, applications, and future directions. Front Nutr. (2025) 12:1636980. doi: 10.3389/fnut.2025.1636980 40771216 PMC12325300

[36] Sharma R Gaur SS Ajay A Shams R Dash KK. Artificial intelligence and precision nutrition: tailoring diets with data-driven insights. Nutrire. (2025) 50:56. doi: 10.1186/s41110-025-00360-4

[37] Massara P Kirkland J Pagani I Huey SL Hirsh H McDonald D . Applying artificial intelligence and machine learning in precision nutrition. Nat Commun. (2026) 17:5880. doi: 10.1038/s41467-026-75004-w 42409828 PMC13338271

[38] Wang X Sun Q. Ultra-processed foods and the impact on cardiometabolic health: the role of diet quality. Diabetes Metab J. (2024) 48:1047–55. doi: 10.4093/dmj.2024.0659 39610133 PMC11621665

[39] Babalola OO Akinnusi E Ottu PO Bridget K Oyubu G Ajiboye SA . The impact of ultra-processed foods on cardiovascular diseases and cancer: epidemiological and mechanistic insights. Aspects Mol Med. (2025) 5:100072. doi: 10.1016/j.amolm.2025.100072

[40] Chang K Gunter MJ Rauber F Levy RB Huybrechts I Kliemann N . Ultra-processed food consumption, cancer risk and cancer mortality: a large-scale prospective analysis within the UK Biobank. EClinicalMedicine. (2023) 56:101840. doi: 10.1016/j.eclinm.2023.101840 36880051 PMC9985039

[41] Liu Z Gao S Yuan Z Yang R Zhang X El-Mesery HS . Exploring formation and control of hazards in thermal processing for food safety. Foods. (2025) 14:2168. doi: 10.3390/foods14132168 40646920 PMC12248888

[42] Chassaing B Koren O Goodrich JK Poole AC Srinivasan S Ley RE . Dietary emulsifiers impact the mouse gut microbiota promoting colitis and metabolic syndrome. Nature. (2015) 519:92–6. doi: 10.1038/nature14232 25731162 PMC4910713

[43] Hu G Flexner N Tiscornia MV L'Abbé MR. Accelerating the classification of NOVA food processing levels using a fine-tuned language model: a multi-country study. Nutrients. (2023) 15:4167. doi: 10.3390/nu15194167 37836451 PMC10574618

[44] Arora N Bhagat S Dhama R Bagler G. Machine learning and natural language processing models to predict the extent of food processing. J Food Compos Anal. (2025) 146:107938. doi: 10.1016/j.jfca.2025.107938

[45] Lemay DG Strohmeier MP Stoker RB Larke JA Wilson SM. Evaluation of large language models for mapping dietary data to food databases. J Nutr. (2026) 156:101678. doi: 10.1016/j.tjnut.2026.101678 42309308

[46] Grilo MF Nunes BS Duran AC Zancheta Ricardo C Baraldi LG Martinez Steele E . Applying the Nova food classification to food product databases using discriminative ingredients: a methodological proposal. Front Public Health. (2025) 13:1575136. doi: 10.3389/fpubh.2025.1575136 40666144 PMC12259438

[47] Fang S Shao Z Mao R Fu C Delp EJ Zhu F . “Single-view food portion estimation: learning image-to-energy mappings using generative adversarial networks,” in *2018 25th IEEE International Conference on Image Processing.* Athens, Greece: IEEE 251–255. (2018) doi: 10.1109/ICIP.2018.8451461

[48] Vinod G He J Shao Z Zhu F. “Food portion estimation via 3D object scaling,” in *2024 IEEE/CVF Conference on Computer Vision and Pattern Recognition Workshops*. Seattle, WA: IEEE (2024). 3741–3749. doi: 10.1109/CVPRW63382.2024.00378

[49] Elbassuoni S Ghattas H El Ati J Shmayssani Z Katerji S Zoughbi Y . DeepNOVA: a deep learning NOVA classifier for food images. IEEE Access. (2022) 10:128523–35. doi: 10.1109/ACCESS.2022.3227769

[50] Chun YL Lo YW Chow C-F Chan CCH Mung SWY. Smart food area–weight system for dietary and nutritional monitoring. IEEE Sens J. (2025) 25:44522–34. doi: 10.1109/JSEN.2025.3620093

[51] Lo YW Mung SWY Chow C-F Chan CCH. IoT-based multiscale weighing system using centralized data aggregation for nutrition intake monitoring. IEEE Sens Lett. (2026) 10:5503204. doi: 10.1109/LSENS.2026.3688480

[52] He Z Mung SWY Kam ACS Chan CCH. Multimodal LLM-driven IoT digital healthcare platform for intelligent dysphagia dietary monitoring. Front Nutr. (2026) 13:1829703. doi: 10.3389/fnut.2026.1829703 42500256 PMC13398057

[53] Paulose-Ram R Graber JE Woodwell D Ahluwalia N. The national health and nutrition examination survey (NHANES), 2021-2022: adapting data collection in a COVID-19 environment. Am J Public Health. (2021) 111:2149–56. doi: 10.2105/AJPH.2021.306517 34878854 PMC8667826

[54] Scarborough P Rayner M Stockley L. Developing nutrient profile models: a systematic approach. Public Health Nutr. (2007) 10:330–6. doi: 10.1017/S1368980007223870 17362528

[55] Bossard L Guillaumin M Van Gool L. “Food-101 – mining discriminative components with random forests,” in *European Conference on Computer Vision.* Zurich: Springer (2014). doi: 10.1007/978-3-319-10599-4_29

[56] Reimers N Gurevych I. “Sentence-bert: sentence embeddings using Siamese BERT-networks,” in *Proceedings of the 2019 Conference on Empirical Methods in Natural Language Processing and the 9th International Joint Conference on Natural Language Processing.* Hong Kong: Association for Computational Linguistics (2019). 3982–3992. doi: 10.18653/v1/D19-1410

[57] Yujian L Bo L. A normalized Levenshtein distance metric. IEEE Trans Pattern Anal Mach Intell. (2007) 29:1091–5. doi: 10.1109/TPAMI.2007.1078 17431306

[58] Li X Sun X Meng Y Liang J Wu F Li J. “Dice loss for data-imbalanced NLP tasks,” in *Proceedings of the 58th Annual Meeting of the Association for Computational Linguistics.* Seattle, WA: Association for Computational Linguistics (2020). 465–476. doi: 10.18653/v1/2020.acl-main.45

